# Communicating COVID-19 exposure risk with an interactive website counteracts risk misestimation

**DOI:** 10.1371/journal.pone.0290708

**Published:** 2023-10-05

**Authors:** Alyssa H. Sinclair, Morgan K. Taylor, Freyja Brandel-Tanis, Audra Davidson, Aroon T. Chande, Lavanya Rishishwar, Clio Andris, R. Alison Adcock, Joshua S. Weitz, Gregory R. Samanez-Larkin, Stephen J. Beckett

**Affiliations:** 1 Department of Psychology and Neuroscience, Duke University, Durham, NC, United States of America; 2 School of City and Regional Planning, Georgia Institute of Technology, Atlanta, GA, United States of America; 3 School of Civil and Environmental Engineering, Georgia Institute of Technology, Atlanta, GA, United States of America; 4 School of Biological Sciences, Georgia Institute of Technology, Atlanta, GA, United States of America; 5 Applied Bioinformatics Laboratory, Georgia Institute of Technology, Atlanta, GA, United States of America; 6 School of Interactive Computing, Georgia Institute of Technology, Atlanta, GA, United States of America; 7 Department of Psychiatry and Behavioral Sciences, Duke University, Durham, NC, United States of America; 8 Department of Neurobiology, Duke University, Durham, NC, United States of America; 9 School of Physics, Georgia Institute of Technology, Atlanta, GA, United States of America; 10 Institut de Biologie, École Normale Supérieure, Paris, France; University of Edinburgh, UNITED KINGDOM

## Abstract

During the COVID-19 pandemic, individuals depended on risk information to make decisions about everyday behaviors and public policy. Here, we assessed whether an interactive website influenced individuals’ risk tolerance to support public health goals. We collected data from 11,169 unique users who engaged with the online COVID-19 Event Risk Tool (https://covid19risk.biosci.gatech.edu/) between 9/22/21 and 1/22/22. The website featured interactive elements, including a dynamic risk map, survey questions, and a risk quiz with accuracy feedback. After learning about the risk of COVID-19 exposure, participants reported being less willing to participate in events that could spread COVID-19, especially for high-risk large events. We also uncovered a bias in risk estimation: Participants tended to overestimate the risk of small events but underestimate the risk of large events. Importantly, even participants who voluntarily sought information about COVID risks tended to misestimate exposure risk, demonstrating the need for intervention. Participants from liberal-leaning counties were more likely to use the website tools and more responsive to feedback about risk misestimation, indicating that political partisanship influences how individuals seek and engage with COVID-19 information. Lastly, we explored temporal dynamics and found that user engagement and risk estimation fluctuated over the course of the Omicron variant outbreak. Overall, we report an effective large-scale method for communicating viral exposure risk; our findings are relevant to broader research on risk communication, epidemiological modeling, and risky decision-making.

## Introduction

Communicating the risk of viral exposure (e.g., for COVID-19 [1], monkeypox [2], or influenza [3]) is crucial for empowering individuals to make informed decisions about everyday risks. For instance, if an individual is anticipating an upcoming trip to attend a wedding, they may be concerned about the risk of getting infected with COVID-19 before or during the trip, potentially disrupting plans and infecting others. The COVID-19 pandemic has led to prolonged, continually evolving public health challenges [1]. Importantly, prior studies have shown that individuals underestimate viral transmission in their communities, leading to risky behaviors [4, 5]. The risk of COVID-19 exposure (and consequently, the risk of excessive cases, hospitalizations, and deaths) can vary substantially across regions and over time. For instance, the emergence of the Omicron variant in the U.S. led to a five-fold increase in national COVID-19 cases in only three weeks (12/20/21–1/10/22) [6]. During the initial Omicron wave, cases and hospitalizations rose, peaked, and declined at different times in different regions. The heterogeneity of this recent outbreak underscores the importance of real-time, local risk information for individuals and policymakers alike. Tailoring decisions to current, local risk levels is crucial for a flexible longer-term pandemic response that addresses public health goals without exacerbating mental health crises [7–9].

Previously, members of our research team developed an interactive website [10] that provides US-county-level COVID-19 risk information (https://covid19risk.biosci.gatech.edu/). The primary goal of the website is to provide real-time, geolocalized risk information that helps individuals and policymakers make decisions that improve public health outcomes. For example, if there is a high risk of encountering SARS-CoV-2 in a particular county, individuals may reduce risk-taking behavior and policymakers may implement public health interventions like mask mandates. Between the launch of the website in July 2020 and the end of the data collection period for the present study (1/22/22), the website was accessed by over 16 million unique users (as identified by aggregate statistics from Google Analytics, which uses browser cookies and user profiles to estimate the number of unique users). Note that due to the discontinuation of our data sources, the website is no longer active. However, an archived version of the website may be accessed at https://covid19risk.biosci.gatech.edu/.

The online dashboard combines documented COVID-19 cases with ascertainment bias information (derived from population-wide serological surveys) to estimate the actual number of COVID-19 infections currently circulating in a region. Using these real-time prevalence estimates, we visualize the risk of SARS-CoV-2 exposure across the U.S. (defined as the probability that one or more individuals at a gathering will be infected with SARS-CoV-2). Users can select different event sizes (ranging from 10 to 5,000 people) to view how risk levels scale with the number of attendees.

Separately, other members of our research team investigated how individuals estimate COVID-19 risks and make decisions [5]. We found that individuals’ perceived risk of COVID-19 was not aligned with actual risk (defined as prevalence-based exposure risk, as described above). Despite being misaligned with reality, perceived risk strongly predicted self-reported compliance with public health guidelines (social/physical distancing, hand washing, etc.) and intentions to participate in risky activities (e.g., dining inside a restaurant, traveling). To realign individuals’ beliefs about risk with reality, we developed a psychological intervention [5, 11]. Our multifaceted intervention combined an imagination exercise (illustrating a specific transmission scenario) with a risk quiz (estimating prevalence-based exposure risk and receiving accuracy feedback). After the intervention, individuals who had been underestimating risk reported increased perceived risk and decreased willingness to take risks, in line with public health goals [5, 11].

In the present study, we implemented elements of this evidence-based intervention in a large-scale online field study. We drew on insights from our prior intervention by adding new interactive elements to the COVID-19 Event Risk website, with the goal of improving individuals’ compliance with public health guidelines. Our interactive risk assessment tools were informed by several theoretical frameworks pertaining to health beliefs, behavior change, and risk literacy. Drawing on the *health belief model* [12], we provided exposure risk information to change *perceived susceptibility*, or beliefs about the likelihood of encountering a health risk or experiencing an adverse health event. Furthermore, we incorporated illustrations and example scenarios to provide specific, concrete information, contextualizing risk across event sizes. Prior studies have shown that concrete examples improve risk estimation accuracy [13], and using specific scenarios and narratives increases the efficacy of risk communication [5, 14, 15]. Lastly, we also designed our website to increase *self-relevance* (by emphasizing current, local risks in everyday scenarios) and *self-efficacy* (by recommending specific risk-mitigating actions, like social distancing and mask-wearing), which have both been shown to be crucial components of persuasion, behavior change, and health communication [16–21].

Several prior papers have offered recommendations for COVID-19 risk communication [22–24], and others have reported expert evaluations of various dashboards for actionable policy decisions [25–28]. To our knowledge, no prior studies have assessed whether data dashboards influence individuals’ risk tolerance and behavioral intentions. Our primary aims were to characterize public perception of COVID-19 exposure risk and assess whether our interactive risk assessment tools could reduce willingness to participate in potentially risky activities.

Prior studies have shown that individuals misestimate health risks (often underestimating risk), especially for probabilistic, abstract, or recurring events [4, 5, 13, 29, 30]. Therefore, we expected that estimates of COVID-19 exposure risk would be variable and often inaccurate. Drawing on our prior findings [5], we expected that participants would tend to underestimate risk on average, but we would observe substantial individual differences in risk estimation—some participants would underestimate risk, whereas others would overestimate risk. On the basis of our prior findings, we also predicted that risk misestimation would differ across event sizes; we expected more severe risk underestimation for larger events.

In the present study, we aimed to measure risk misestimation and test whether our interactive website tools would change risk-taking intentions. Here, we report data from 11,169 unique users who interacted with the COVID-19 Event Risk website during a 4-month period (9/22/21–1/22/22). In brief, we found that interacting with the COVID-19 Event Risk website reduced willingness to participate in risky events. This increase in risk aversion was most prominent for users who had been underestimating risk and users who viewed information about larger event sizes. Importantly, even among participants who voluntarily sought information on our website (i.e., individuals who are likely already aware of or interested in COVID risks), we observed substantial risk misestimation, demonstrating the importance of intervention. We also observed a political divide in engagement with COVID-19 information, underscoring the need for new strategies to communicate risk to conservative individuals. Overall, our results suggest that communicating local, contextualized risk information with interactive tools may shift individuals’ risk tolerance and decrease the likelihood of viral transmission. With these insights, we offer concrete recommendations for risk communication to improve public health outcomes.

## Materials and methods

### Participants

We collected data from 11,169 unique users who interacted with the COVID-19 Event Risk Tool (covid19risk.biosci.gatech.edu) during a 4-month period (9/22/21–1/22/22). We identified unique users by public IP addresses. Using a modal dialog box, we asked participants to assert that they were ≥18 years of age, residing in the U.S., and agreed to share their responses for research purposes. During the data collection period, the website received approximately 247,000 visits from approximately 170,000 unique users. However, not all participants provided responses to all interactive elements of the website; sample sizes differed across analyses (S1 and S2 Tables). We saved and analyzed data from all users (N = 11,169) who agreed to share their data and interacted with the website tools.

During the data collection period, we also conducted several advertising campaigns on social media, intended to increase traffic to the Event Risk Tool website (S3 Table). Most of these advertising campaigns targeted a general audience (all users aged 18+ years, currently residing in the United States), though some campaigns specifically targeted politically-conservative individuals. In total, these campaigns generated 3,663 clicks (users redirected to the Event Risk Tool website) between 11/8/21 and 1/1/22. The website was also featured 15 times in various local and national news sources during the data collection period (S4C Fig).

The study was approved by the Duke University Campus Institutional Review Board (protocol #2022–0010) and the Georgia Institute of Technology Institutional Review Board (protocol #H21292). The requirement for formal informed consent was waived by both ethics committees. Although this convenience sample was subject to self-selection bias (i.e., participants voluntarily sought out information about COVID-19 risks), we observed substantial risk misestimation in this sample, demonstrating that correcting risk perception was indeed necessary in this population.

### Risk map

**Fig 1:**
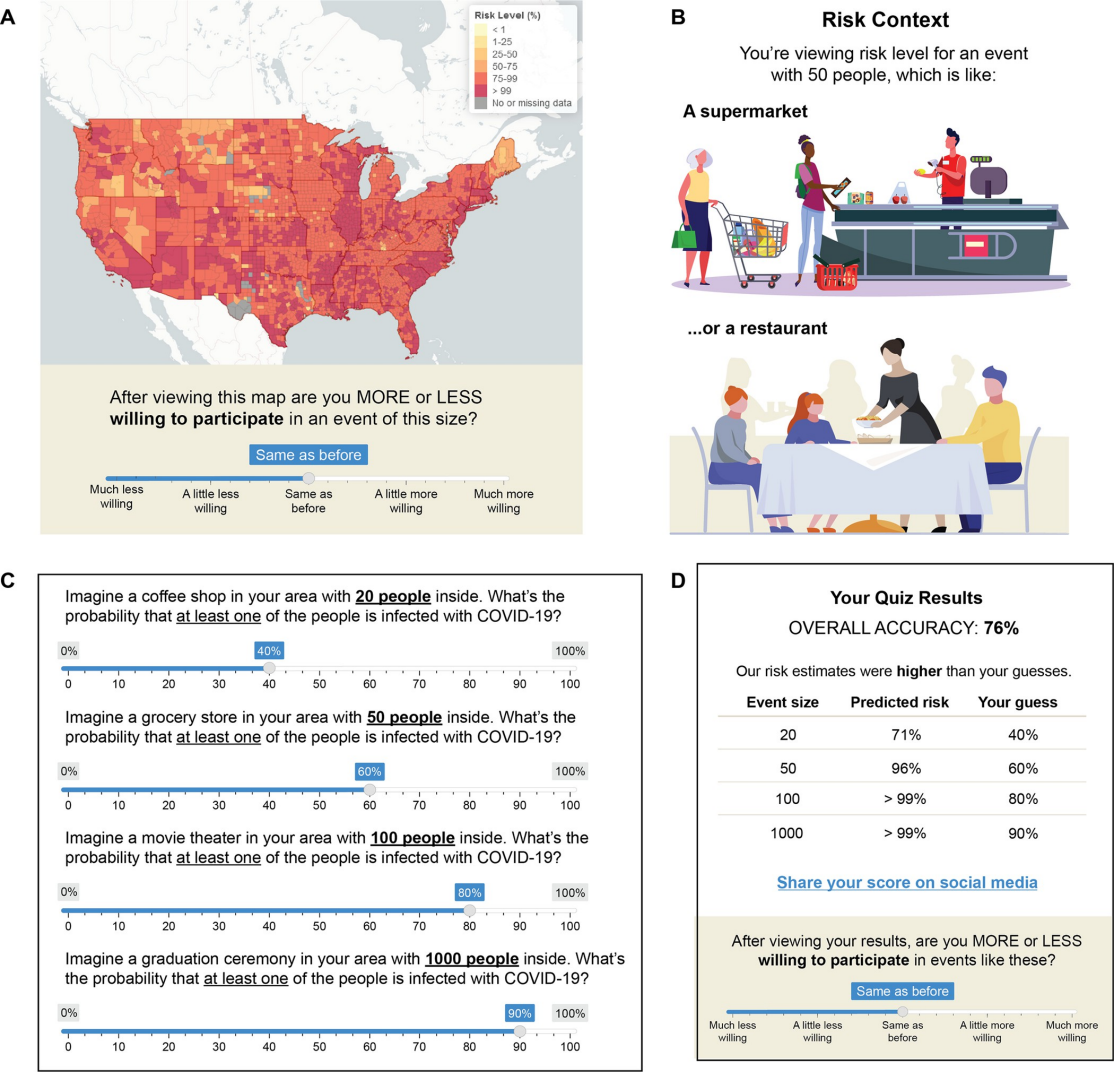
Overview of website features. A) The homepage of the website displays a map of the USA with individual counties color-coded by exposure risk level. The map depicted is for January 25, 2022 with an ascertainment bias of 4 and a 50-person event size. Below the map is a willingness rating. B) Adjacent to the map is a “risk context” panel that contextualizes the selected event size with two example scenarios. Illustrations adapted from freepik.com under a CC BY license, with permission from FreePik, original copyright 2023. C) On a separate page of the website, the risk quiz enabled users to test their knowledge of current risk levels in their own local communities. D) After submitting risk quiz responses, users viewed a feedback box and willingness rating.

On the homepage of the website, participants viewed a county-level map of the United States that displayed the risk level of attending an event, given the event size and location (Fig 1A). To estimate the actual number of COVID-19 cases (accounting for under-testing), we combined statistics about documented COVID-19 cases with ascertainment bias information derived from population-wide serological surveys [10]. We defined exposure risk as the estimated probability (ranging from <1% to >99%) that at least one individual at an event of a given size is infected with SARS-CoV-2. Details about risk calculation, website development, and deployment are described in a prior report [10].

Next to the risk map, we displayed a “risk context” panel (Fig 1B) that contextualized the selected event size with real-world examples and illustrations (e.g., “You’re viewing risk levels for an event with 50 people, which is like a supermarket or a restaurant.”). Participants were able to interact with the map by selecting various event sizes (ranging from 10 people to 5,000 people). Below the map was a survey question with a 5-point rating scale: “After viewing this map, are you MORE or LESS willing to participate in an event of this size?” (1 = *Much less willing … 5 = Much more willing*). Note that we only obtained a single measure of “change in willingness” (as opposed to separate before-and-after measures of absolute willingness) because of feasibility and user experience constraints (Fig 1A and 1D).

### Risk quiz

On a separate page of the website, we included an interactive risk quiz that allowed participants to test their own knowledge of local risk levels and receive accuracy feedback (Fig 1C). After selecting a location, participants answered four questions about various scenarios within their own local communities. The first question stated, “Imagine a coffee shop in your area with **20 people** inside. What’s the probability that at least one of the people is infected with COVID-19?”. Participants estimated the exposure risk probability by using a sliding scale from 0% to 100%. The following three questions on the risk quiz followed the same format but varied the scenario and event size (grocery store with 50 people; movie theater with 100 people; graduation ceremony with 1,000 people).

After submitting their quiz responses, participants viewed a feedback window (Fig 1D) that displayed an overall accuracy score and summary statement (e.g., “Overall Accuracy: 75%. Our risk estimates were higher than your guesses.”). We also displayed a table showing the user’s guess and our prevalence-based risk estimate for each event size. Below the table, we included a 5-point rating scale similar to the scale shown below the Risk Map: “After taking this quiz, are you MORE or LESS willing to participate in events in your area?” (1 = *Much less willing … 5 = Much more willing*).

The slight majority of participants (56%) played the risk quiz only a single time. However, some participants played the risk quiz multiple times (e.g., for multiple locations or on different days). We limited our analyses to the first risk quiz completed by each user because the first quiz offers insight into an individual’s risk estimation bias prior to receiving feedback. Additionally, we excluded risk quizzes submitted with all default values (16% of users who submitted a response of 50% for every question). We ensured that these decisions to exclude data did not meaningfully change the results (S1 Text).

### Demographic survey

To collect more information about our user base, we included a brief demographics survey hosted by Qualtrics. Survey data collection occurred between 11/17/21 and 1/22/22. Participants who completed the optional survey (N = 612) had the opportunity to enter a monthly lottery draw for a $50 gift card. The demographics survey included questions about age, gender, political affiliation, vaccination status, and individual experiences during the COVID-19 pandemic. Results from the demographics survey are provided in the Supporting Information (S3 Text).

Additionally, we used leveraged county-level demographic information from the risk quiz. The website tool attempted to automatically detect each participant’s location from browser data, but participants were able to manually select their location if automatic detection failed. We integrated this location information from the risk quiz with county-level data from several sources: The New York Times U.S. COVID-19 database [6, 31], the 2019 American Community Survey (5-year estimates) from the U.S. Census Bureau [32], and presidential election data from the MIT Election Data and Science Lab [33].

### Statistical analysis and data sharing

All analyses were conducted in R (v.4.1.1) with RStudio (v.2021.09.0). See Supporting Information (S1 Text) for details. Statistical significance was evaluated with two-sided tests and a threshold of *p* < 0.05. Continuous variables were standardized and mean-centered before inclusion in regression models (*z*-scored); however, all figures depict results in original units for ease of interpretation. For analyses that included multiple observations from each participant, we used mixed-effects models with random intercepts for participants. For risk quiz analyses, which were associated with accurate location data, we also included random intercepts for different U.S. counties, because different counties may differ in political leaning, pandemic restrictions and policies, COVID-related attitudes, population density, and population immunity. These factors may contribute to shared variance among multiple observations from a given county.

All data and code are publicly available in a permanent repository hosted by the Open Science Framework (DOI: 10.17605/OSF.IO/MBH9W). To protect the privacy of our participants, some identifiers have been removed from the raw data (the last two digits of IP addresses and open-ended personal comments from the demographics survey). All code and data sources for the COVID-19 Event Risk website (https://covid19risk.biosci.gatech.edu/) are publicly available on the About page. The study was not preregistered.

## Results

### Risk map: Change in willingness to participate in public gatherings

First, we investigated whether participants reported being less willing to participate in events that could contribute to the spread of COVID-19 after viewing the US risk map. Responses to the rating scale below the risk map indicated that on average, participants reported being significantly less willing to participate in events after viewing the map (mean change in willingness = -0.41 points, *t*_*(*8576)_ = -35.87, *p <* 0.0001, Cohen’s *d* = -0.39, 95% CI [-0.41, -0.37]).

**Fig 2:**
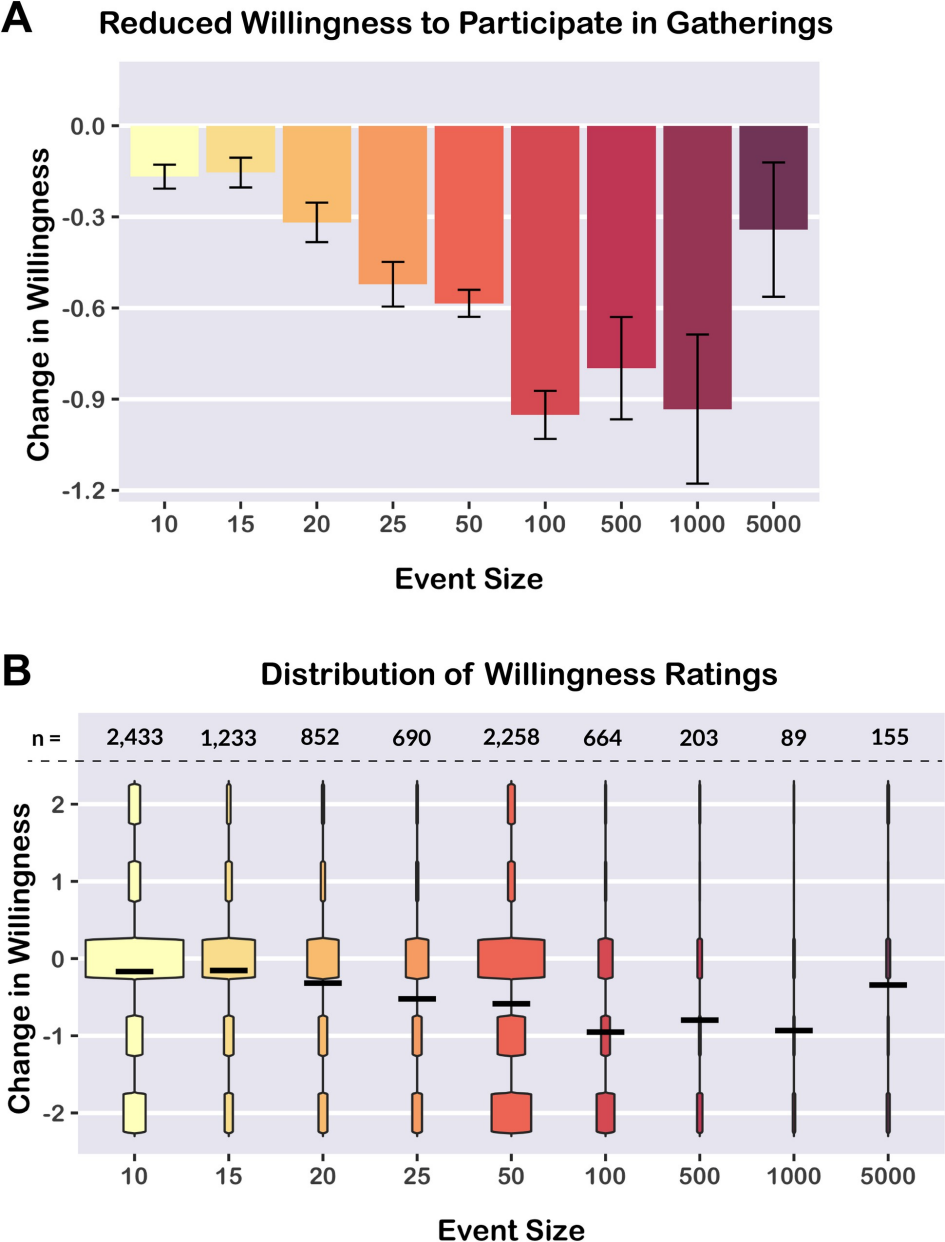
Reduced willingness to participate in large events. Participants reported being significantly less willing to participate in events of a given size after viewing the corresponding risk map. The decrease in willingness was greater for larger event sizes, with the exception of the largest event size (5,000 people), likely because participants were already unwilling to participate in very large events. Change in willingness was rated on a 5-point Likert-style scale. A) Mean change in willingness by event size. Error bars indicate 95% confidence intervals. B) Violin plots depicting data distributions. Horizontal bars indicate means. Numbers above the dotted line indicate the number of observations per event size.

Visualizing change in willingness across event sizes revealed a non-linear relationship (Fig 2A). Therefore, we used a polynomial mixed effects regression model (including random intercepts for unique participants) to predict *change in willingness* to participate in public gatherings (standardized) from *event size* (a continuous numerical variable, log-transformed and standardized), including a quadratic term for event size to account for non-linearity (orthogonalized). We found that event size was significantly associated with change in willingness (linear term: β = -16.64, 95% CI [-18.63, -9.87], *t* = -14.66, *p <* 0.0001; quadratic term: β = 13.81, 95% CI [11.81, 15.81], *t* = 13.55, *p <* 0.0001). Overall, viewing the U.S. risk map for larger event sizes led to significantly greater decreases in willingness to participate in those events, relative to smaller event sizes (Fig 2A and 2B). The exception to this pattern was for the largest event bin (5,000 people), which was associated with a modest decrease in willingness. The most likely explanation for this non-linear pattern is that most participants were already unwilling to participate in an event with 5,000 people, resulting in a smaller decrease in willingness. A table of descriptive statistics for willingness ratings across event sizes is provided in the Supporting Information (S1 Table).

### Risk quiz: Misestimation of risk

Using data from the risk quiz, we tested whether participants misestimated COVID-19 exposure risk. For each participant, we calculated *risk estimation error* for each event size by subtracting our prevalence-based measure of exposure risk from the participant’s guess. This sign of the risk estimation error score indicates the direction of misestimation (positive values indicate risk overestimation, whereas negative values indicate risk underestimation). The magnitude of this error measure indicates the severity of risk misestimation. On average (across all event sizes), there was a small bias towards underestimation of risk, consistent with our prior research (mean risk estimation error = -2.49 points, *t*_(4840)_ = -6.67, *p <* 0.0001, Cohen’s *d* = -0.10, 95% CI [-0.12, 0.07]) [5]. Importantly, however, there was substantial risk misestimation in both directions—although the slight majority of participants underestimated risk, many overestimated risk. As a result, the average error score is close to 0 (indicating accurate estimation), despite heterogeneity in individual risk estimates. This distribution of risk estimates closely aligns with our prior findings from an intervention study [5]. Interestingly, despite the correct answers being provided elsewhere on the website (the risk map), we still observed wide variability in risk estimation.

**Fig 3:**
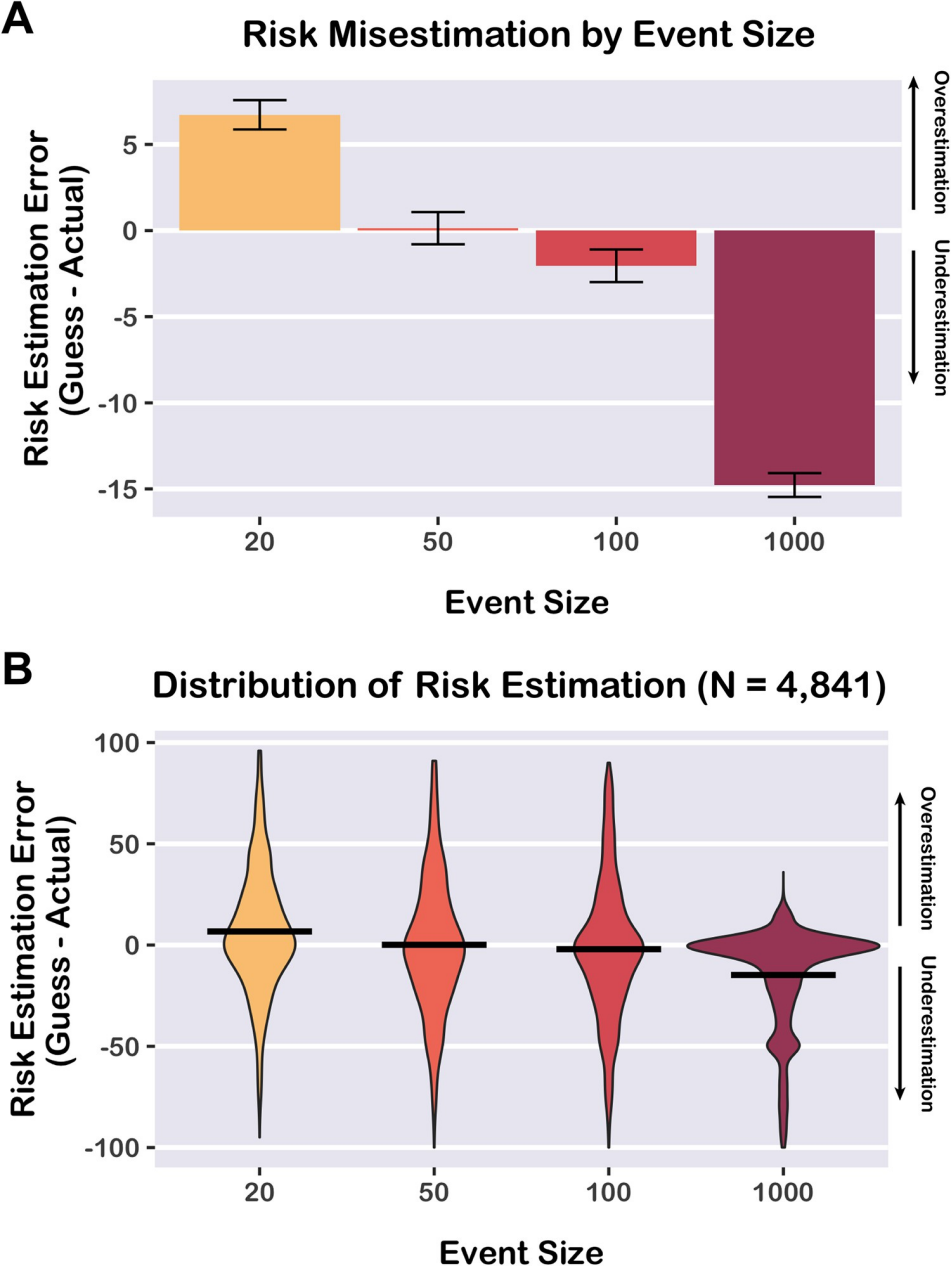
Risk misestimation by event size. Average risk estimation error (participant’s guess–prevalence-based risk estimate) differed across event sizes. Participants tended to overestimate the risk of small events (20 people) but underestimate the risk of large events (100 and 1,000 people). Plots depict results from 4,841 participants who completed the risk quiz. A) Mean risk estimation error by event size. Error bars depict 95% confidence intervals. B) Violin plots depicting data distribution. Horizontal bars indicate means.

Using linear mixed effects regression (including random intercepts for participants and counties), we tested whether risk estimation error differed across the four event sizes tested on the quiz (a factor variable with levels for event sizes 20, 50, 100, and 1,000). There was a significant effect of event size on risk estimation error, *F*_(3,14520)_ = 1123.3, *p <* 0.0001. Participants tended to overestimate the risk of small events (20 people) but underestimate the risk of large events (100 and 1,000 people) (Fig 3A and 3B). All pairwise contrasts between event sizes were significant (*p* < 0.0001) after correcting for multiple comparisons with Tukey’s HSD (S2 Table).

Note that risk estimation for large events can be subject to a ceiling effect, which can make it impossible to overestimate risk. In a control analysis, we tested a subset of data (*n* = 501) in which the predicted risk for an event with 1,000 people was ≤ 90% (i.e., making overestimation possible) and found that there was still a significant tendency to underestimate risk for this event size (mean risk estimation error = -8.41 points, *t*_(500)_ = -6.52, *p* < 0.0001, *d* = -0.29, 95% CI [-0.38, -0.20]).

### Risk quiz: Change in willingness

Next, we tested whether engaging with the risk quiz (and viewing accuracy feedback) changed willingness to participate in events. On average, participants reported being less willing to participate in events after viewing the feedback about their own accuracy, *t*_(2217)_ = -17.66, *p <* 0.0001, *d* = -0.38, 95% CI [-0.42, -0.33].

Finally, we investigated whether participants’ risk estimation bias predicted change in willingness to participate in events. For each participant, we averaged the signed risk estimation error scores across the four event sizes to obtain a summary metric of *risk estimation error*; negative values indicate a tendency towards risk underestimation, whereas positive values indicate risk overestimation. Using linear mixed effects regression (including random intercepts for counties), we predicted *change in willingness* from *risk estimation error* (continuous variable). There was a significant effect of risk estimation error on change in willingness to participate in potentially risky events, β = 0.26, 95% CI [0.22, 0.31], *t* = 10.78, *p <* 0.0001. In other words, participants who underestimated risk were more likely to report decreases in willingness to take risks after receiving feedback from the risk quiz. More severe risk underestimation was associated with greater decreases in willingness to take risks.

**Fig 4:**
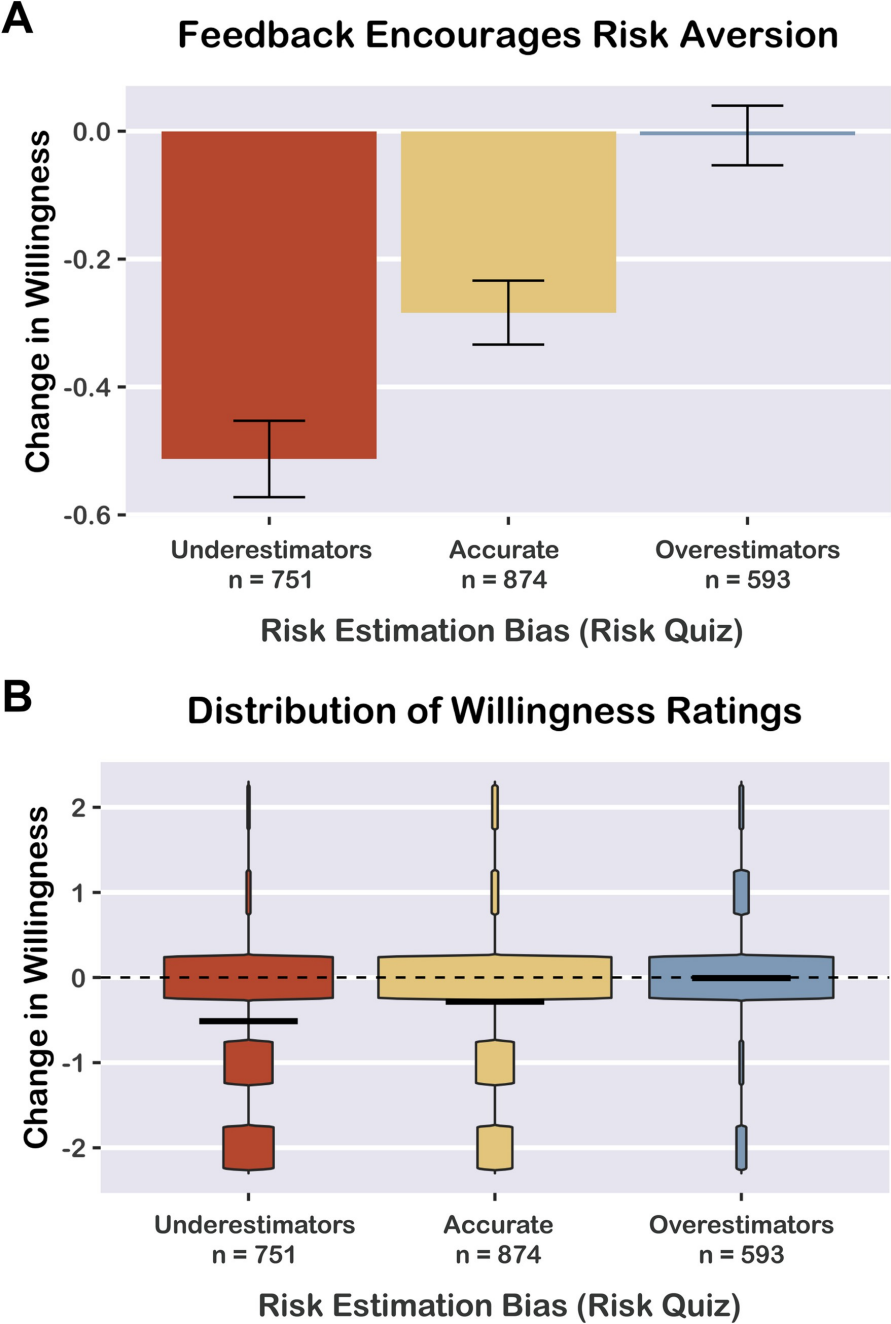
Feedback encourages risk aversion. For visualization purposes, we classified participants as Overestimators, Accurate Estimators, or Underestimators based on their average risk estimation error score. Overestimators reported no significant change in willingness to participate in events after viewing feedback about their risk estimation accuracy. Accurate Estimators showed a moderate decrease in willingness, and Underestimators showed the largest decrease in willingness. A) Mean change in willingness by risk estimation type. Error bars depict 95% confidence intervals. B) Violin plots depicting data distribution. Horizontal bars indicate means. Dotted line indicates zero, no change in willingness.

For the sake of visualization, we classified participants into three groups according to their risk estimation error scores (Fig 4A and 4B). We classified participants as either *Risk Underestimators* (average error > +10; *n* = 1,773 total; *n* = 751 with willingness data), *Accurate Estimators* (average error between -10 and +10; *n* = 1,722 total; *n* = 874 with willingness data), or *Risk Overestimators* (average error < -10; *n* = 1,346 total; *n* = 593 with willingness data). Underestimators showed the greatest decrease in willingness (*M* = -0.51, *t*_(750)_ = -16.85, *p <* 0.0001, *d* = -0.61, 95% CI [-0.69, -0.54]), Accurate Estimators showed a smaller decrease in willingness (*M* = -0.28, *t*_(873)_ = -11.13, *p <* 0.0001, *d* = -0.38, 95% CI [-0.45, -0.31]), and Overestimators showed no significant change in willingness (*M* = -0.01, *t*_(592)_ = -0.28, *p* = 0.777, d = -0.01, 95% CI [-0.09, 0.07]).

### Risk quiz: Political partisanship

We investigated political disparities in user engagement by leveraging location data from risk quiz submissions. Using county-level demographic information, we investigated whether user participation and responsiveness differed depending on political leaning. On the basis of a survey we administered (S3 Text) and prior studies that have investigated political differences in COVID-19-related attitudes and information seeking [34–36], we hypothesized that individuals from conservative-leaning counties would be less likely to visit the website and use the interactive tools. To investigate these questions, we leveraged location data to identify the political leaning of each county, determined by the proportion of votes for the Democratic or Republican parties in the 2020 election. Note that this approach leverages location data to draw inferences about politics, which does not necessarily reflect individual attitudes—it is possible that users from conservative-leaning counties were politically liberal or moderate. However, living in a conservative county may also influence one’s beliefs about COVID-19 regardless of political beliefs, due to differences in local public health policies, messaging, and social norms.

**Fig 5:**
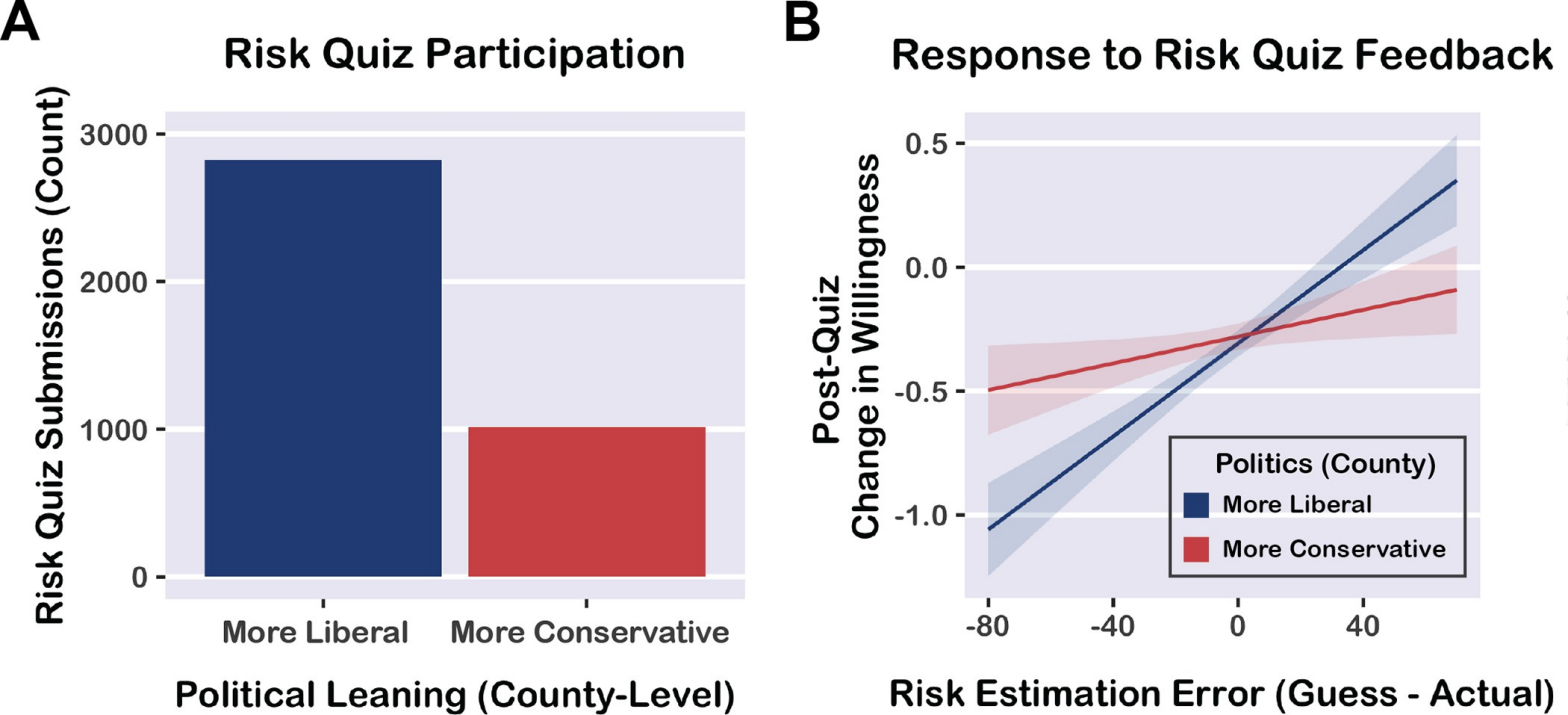
A) More risk quizzes were submitted for liberal counties (defined as greater vote share for the Democratic party than the Republican party in the 2020 presidential election) than for conservative counties (greater vote share for the Republican party), suggesting a political divide in user engagement. B) Predicted values from a linear mixed effects regression model predicting post-quiz change in willingness from *risk estimation error* (averaged across event sizes for each user) and county-level political leaning (% vote for the Republican party, a continuous variable). Blue and red lines depict -1 SD and +1 SD levels of the political leaning variable respectively, dichotomized for visualization only. The intervention was less effective for participants from more conservative counties (i.e., attenuated effect of risk quiz feedback on change in willingness). Shaded bands indicate 95% confidence intervals.

First, we identified liberal and conservative counties according to the majority vote for either the Democratic or Republican party in the 2020 presidential election. We found that 2.8 times more risk quizzes were submitted from liberal-leaning counties than conservative-leaning counties, indicating a strong bias in user participation, χ^2^ (1, *N* = 3837) = 855.71, *p* < 0.0001, expected proportion = 0.50, observed proportion = 0.74, 95% CI [0.72, 0.75] (Fig 5A).

Next, we tested whether risk misestimation was influenced by county-level political leaning. Using linear mixed effects regression (including random intercepts for counties), we predicted *risk estimation error* scores (averaged across event sizes for each individual) from *political leaning* (% vote share for the Republican party, continuous variable). We also included covariates for the total number of voters and contemporaneous COVID-19 prevalence in each county (new cases in the past 14 days, per 100,000 people). All parameter estimates are reported in S4 Table. There was no significant effect of county-level political leaning on risk estimation error, *β* = -0.03, 95% CI [-0.07, 0.01], *t* = -1.52, *p* = 0.131. In other words, county-level political leaning did not significantly predict risk misestimation. One possibility is that participants from liberal- and conservative-leaning counties are equally aware of COVID-19 risks (or equally inaccurate at estimating risk), but show differences in attitudes, risk tolerance, and behavior. Alternatively, this null effect may be due to the limitations of our political leaning variable (i.e., participants from conservative counties may actually be conservative, liberal, or moderate), or reflect the overall variability and inaccuracy of risk estimation.

We then investigated whether political leaning was related to the efficacy of the website tools. Using linear mixed effects regression (including random intercepts for counties), we predicted *change in willingness* after the risk quiz from *political leaning*, *risk estimation error* (averaged across event sizes for each individual), and the interaction term. As described above, the model also included covariates for the total number of voters and COVID-19 prevalence. There was a significant interaction between political leaning and risk estimation error (*β* = -0.10, 95% CI [-0.15, -0.05], *t* = -4.01, *p* < 0.0001), demonstrating that the effect of risk estimation error (i.e., quiz feedback) on willingness to take risks was weaker for participants from conservative counties (Fig 5B). In other words, our risk assessment tools were more effective for participants from liberal counties and less effective for participants from conservative counties. There was a weak main effect of political leaning on change in willingness, driven by the interaction described above (*β* = 0.05, 95% CI [0.00, 0.10], *t* = 1.96, *p* = 0.051). There was also a significant main effect of risk estimation error on change in willingness (*β* = 0.18, 95% CI [0.13, 0.24], *t* = 7.14, *p* < 0.0001), demonstrating the overall efficacy of our risk assessment tools. All parameter estimates are reported in S5 Table.

Lastly, we also tested whether local public health restrictions (face mask policies) predicted change in willingness, as these restrictions may differ systematically across liberal and conservative counties, and in turn influence individuals’ willingness to participate in public gatherings. Local mask-wearing policies did not predict change in willingness, nor did mask-wearing policies interact with political leaning (S2 Text). We also conducted exploratory demographic analyses with the survey data; there were no significant effects of political attitudes, age, or gender on either risk estimation accuracy or change in willingness (S3 Text).

### Risk quiz: Characterizing changes over time

**Fig 6:**
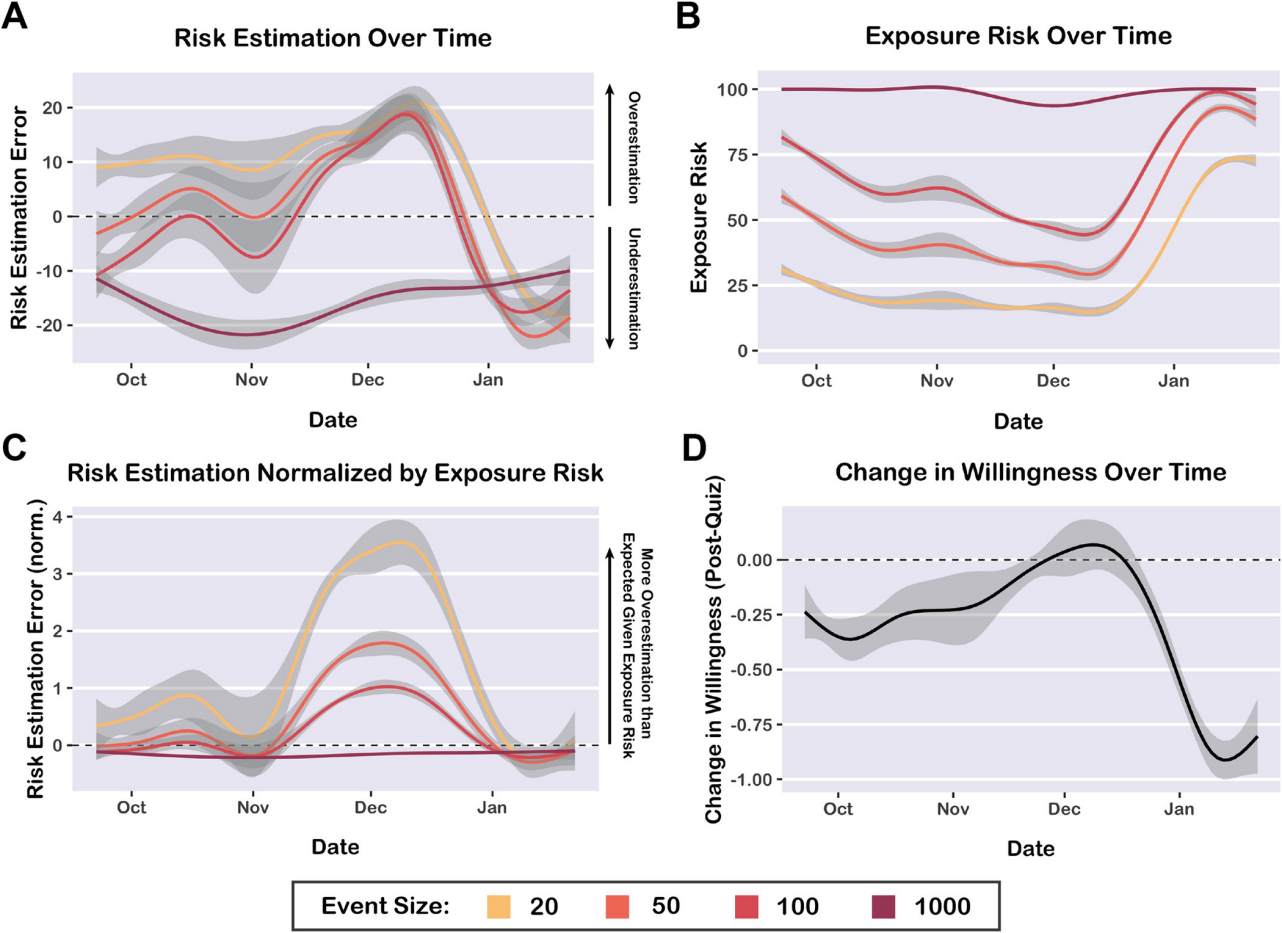
Temporal analysis of risk quiz data. A) Average risk estimation error (guess–actual) for each event size assessed in the risk quiz, plotted over the course of the 4-month data collection period. Participants consistently underestimated risk for the largest event size (1,000 people), whereas risk misestimation fluctuated over time for the other event sizes. Dotted line at *y* = 0 indicates accurate risk estimation. B) Prevalence-based exposure risk paralleled risk estimation accuracy over time, demonstrating that risk perception is related to prevalence. Participants tended to overestimate risk when prevalence was low, but underestimate risk when prevalence was high. C) Risk estimation error scores (as seen in Panel A) normalized by exposure risk (as seen in Panel B) for each county and timepoint. Dotted line at *y* = 0 indicates that risk misestimation is as-expected given the prevalence-based exposure risk. Participants overestimated risk for small-to-medium event sizes during November and December of 2021. D) Change in willingness to take risks (after the risk quiz) plotted over time. Dotted line at *y* = 0 indicates no change in willingness. Participants reported the greatest decreases in willingness during the Omicron wave, from December through January. Gray bands indicate 95% confidence intervals.

Data collection took place over a 4-month period that included the emergence and rise of the Omicron variant, as well as several major U.S. holidays (Thanksgiving, Christmas, and New Year’s Eve). Using data from the risk quiz, we visualized fluctuations in risk misestimation and change in willingness over time. Temporal trends for risk misestimation differed across event sizes (Fig 6A). For the largest event size (1,000 people), participants consistently underestimated risk throughout the 4-month period. For the other three event sizes assessed in the quiz (20, 50, and 100 people), participants fluctuated between periods of risk overestimation (particularly during November and December 2021) and periods of risk underestimation (particularly during the height of the Omicron wave in January).

However, it is important to note that risk misestimation was also significantly associated with prevalence-based exposure risk (*β* = -0.68, 95% CI [-0.72, -0.64], *t* = 34.41, *p* < 0.0001). Participants tended to underestimate risk when prevalence was high, but overestimate risk when prevalence was low. Prevalence-based exposure risk fluctuated considerably throughout the 4-month data collection period, reflecting the decline of the delta variant wave and the rise of the Omicron variant wave (Fig 6B).

Therefore, we investigated whether risk misestimation differed over time, after accounting for the effect of prevalence on risk misestimation. We normalized the timeseries data for risk misestimation by dividing the average risk estimation score (per timepoint, per county) by the prevalence-based COVID-19 exposure risk score for each event size (Fig 6C). In this normalized plot, a score of zero indicates that the degree of risk misestimation was as-expected *given the prevalence* of COVID-19 for a given place and time. Importantly, in this plot, zero does not necessarily indicate accurate risk estimation (refer to Fig 6A to assess risk estimation accuracy). The normalized data revealed that during November and December of 2021, participants tended to overestimate risk for small-to-medium event sizes (20, 50, and 100 people), above and beyond the variance in risk misestimation that can be explained by prevalence. This period of risk overestimation may be explained by concern over the recently-identified Omicron variant, anticipation of a winter outbreak, and preparation for holiday travel and gatherings. In line with this explanation, the COVID-19 Event Risk website received peak press coverage and user traffic around the Thanksgiving holiday (S4 Fig).

We also examined change in willingness (after viewing risk quiz feedback) over time (Fig 6D). Participants tended to report greater decreases in willingness during the Omicron wave (December and January), highlighting the benefits of communicating risk during outbreaks. Overall, our temporal analyses revealed that public perception of COVID-19 exposure risk fluctuates over time, likely influenced by disease prevalence, media coverage, and holidays.

## Discussion

Realigning perceived risk with actual risk is crucial for empowering individuals to make everyday health decisions during the pandemic. In this large-scale risk communication study, we assessed whether an interactive website influenced individuals’ risk tolerance to support public health goals. We found that after learning about the risk of COVID-19 exposure, participants reported being less willing to participate in potentially risky events. This increase in risk aversion was especially pronounced for large event sizes and for individuals who had been underestimating risk, demonstrating that our risk assessment tools were effective at changing risk-taking intentions to potentially reduce COVID-19 transmission. Leveraging county-level demographic information, we showed that individuals from liberal-leaning counties were more likely to use our risk assessment tools and report decreases in willingness to take risks. Overall, our results demonstrate the beneficial impact of large-scale risk communication platforms, while highlighting the need for new strategies to bridge the political divide in COVID-19 information consumption and attitudes.

### Counteracting underestimation and encouraging caution

Participants reported being less willing to participate in events after viewing the risk map. Event size showed a curvilinear relationship with change in willingness: Within the range of event sizes from 10 to 1,000 people, participants reported greater decreases in willingness for larger event sizes. However, the largest event size (5,000 people) was associated with only a moderate decrease in willingness, likely because most participants were already quite unwilling to participate in such a large event. Overall, the risk map data suggests that public health messaging during the COVID-19 pandemic may be most impactful by cautioning against moderately-large events ranging from 25 to 1,000 people (e.g., restaurants, weddings, concerts). Risk statistics are also most informative for events in this size range; very small events typically have low risk of COVID-19 transmission, whereas very large events typically have risk levels close to 100% [10] (S1 Fig).

Results from the risk quiz revealed that participants tended to overestimate the risk associated with small events (20 people), but underestimate the risk associated with large events (100 and 1,000 people). Prior studies on cognitive biases have reported that people tend to misestimate risk, especially for rare events [30, 37–39]. Our results enrich this literature by showing that the direction and magnitude of risk misestimation depends on event size.

Overall, we observed a wide range of risk misestimation, with some participants overestimating risk and others underestimating risk. On average, there was a slight bias towards risk underestimation, but the average error score was close to zero (indicating accurate estimation), likely in part because the correct answers were available on the risk map page of the website. Although the group average was relatively accurate, individual participants demonstrated a wide range of risk misestimation in both directions. In the present study, we were primarily interested in how individuals correct beliefs and behavioral intentions after receiving feedback about their own risk misestimation, as opposed to population-wide risk estimation biases. Our findings align with prior studies on the “wisdom of the crowd,” which have shown that averaging variable and inaccurate estimates from many individuals can produce remarkably accurate estimates [40–42].

We also found that completing the risk quiz (and receiving accuracy feedback) decreased willingness to participate in events that could spread COVID-19. The effect of the risk quiz on willingness differed depending on participants’ baseline misestimation bias: Risk Underestimators showed the greatest average decrease in willingness after receiving feedback, Accurate Estimators showed a smaller decrease in willingness, and Risk Overestimators showed no change in willingness. Other prior studies found that beliefs about COVID-19 risks predict compliance with public health guidelines and risk-taking behavior [5, 43–46]. For instance, individuals tend to underestimate the exponential nature of COVID-19 transmission; correcting this misperception increases support for social distancing [47]. Therefore, correcting risk misestimation and decreasing willingness to take risks offers benefits for public health. However, excessive risk aversion may contribute to anxiety, distress, and pandemic fatigue [8, 9, 48, 49]; an important goal for public health communication is to correct risk misestimation in both directions, as in our prior intervention study [5].

Interestingly, our data distributions (as shown in Fig 2B and 4B) indicated that many participants did not report any change in willingness, but others reported substantial changes in willingness. Although average changes in willingness were small in absolute terms (between -0.2 and -0.9 points on the 5-point willingness scale), these point estimates result from averaging data from responders and non-responders. The risk map results demonstrated that communicating the risk associated with larger event sizes (Fig 2B) increased the number of participants who responded to our risk assessment tools. The risk quiz results indicated that changes in willingness depended on baseline risk misestimation (Fig 4): participants who underestimated risk were the most likely to report decreases in willingness after receiving feedback. Conversely, individuals who had overestimated risk or accurately estimated risk were less likely to report any change in willingness. Understanding why some individuals do not respond to risk information and developing strategies that elicit responses from more individuals are important goals for future research.

A limitation of the present study is that due to feasibility and user experience constraints, we only collected a single measure of change in willingness to participate in potentially-risky gatherings. However, in our prior intervention study [5], we collected separate measurements of willingness pre- and post-intervention; these data offer additional insight into risk tolerance. In this prior study, participants rated willingness to engage in 15 different everyday activities (if hypothetically all public health restrictions were lifted), using a 5-point scale (1 = *definitely would not do this* … 5 = *definitely would do this*). Data from this previous study indicated that at baseline, participants were moderately willing to engage in smaller, relatively safer activities like an outdoor picnic with friends (mean willingness rating = 3.10) or retail shopping while wearing a mask (3.51), moderately willing to participate in medium-sized gatherings like indoor restaurant dining (2.38), and least willing to participate in large, crowded gatherings like flying on an airplane (1.94), or attending a house party (1.63). It is likely that willingness to take risks was greater in the present study, as we collected data more than a year after the prior intervention study, when most public health restrictions had been lifted.

Overall, our findings demonstrate that online, interactive risk assessment tools can effectively communicate current and local information about health risks. The *health belief model* describes several key factors that influence how individuals make health decisions: perceived susceptibility, perceived severity, and perceived benefits [12]. Our study targeted perceived susceptibility, here defined as exposure risk. Combining information about exposure risk with information about severity (i.e., the likelihood of severe disease for yourself and close others) and cost-benefit tradeoffs can help individuals make decisions. The design of the Event Risk Tool website was informed by prior studies on risk communication and behavior change, which have shown that self-relevance and specificity improve belief updating, risk estimation, and persuasion [5, 11, 13, 16, 18–20]. Additionally, our interactive tools and concrete guidance (i.e., providing actionable information about everyday activities) may increase perceived self-efficacy, which influences health decisions and behavioral intentions [22].

### User demographics and temporal effects

We explored user demographics by leveraging geographical information and conducting a survey. Approximate location data indicated that users from across the U.S. engaged with the website (S2 Fig). Using location data from the risk quiz, we identified political differences in user engagement. There were 2.8 times more risk quizzes submitted from liberal-leaning counties than conservative-leaning counties, suggesting a substantial political divide in COVID-19 information seeking. Importantly, the efficacy of our risk assessment tools also differed depending on political leaning: The risk quiz feedback beneficially influenced willingness to take risks for participants from liberal counties but was less effective for participants from more conservative counties.

However, county-level political leaning did not significantly predict risk estimation accuracy. There are several reasons why we may not have observed an effect of county-level political leaning on risk estimation accuracy. In general, we found that risk estimation was variable and inaccurate; it may be that participants from liberal- and conservative-learning counties alike struggle to estimate risk without showing systematic biases towards either underestimation or overestimation. Another important consideration is that participants from conservative-leaning counties might be conservative, liberal, or moderate. Furthermore, all users who voluntarily sought COVID-19 risk information on our website were likely risk-aware and interested in learning more about COVID-19; although we observed considerable risk estimation in this sample, the overall bias towards risk underestimation was slight.

Additionally, we conducted a demographics survey that supported and expanded on these geographically-driven inferences. Survey responses indicated that users of all ages engaged with the website (ranging from 18 to 88), but our sample was biased towards politically liberal individuals (71%) and women (67%) (S3 Text). The proportion of self-identified liberal individuals on the survey (71%) closely aligned with the proportion observed in the county-level political data (74%), supporting the geographical inference. Exploratory demographic analyses with the survey data did not reveal any effects of political attitudes, age, or gender on either risk estimation accuracy or change in willingness (S3 Text).

Our results support the idea that liberals are more likely to seek out risk information and more willing to change their risk tolerance when presented with feedback about their risk misestimation. These partisan effects underscore the importance of targeted risk communication to reach broader audiences and counteract biases. Recent studies have demonstrated that political partisanship has contributed to dramatic differences in risk perception and trust [34, 36], quantity and quality of information seeking [36, 50], misunderstanding of viral transmission [47], behavioral responses to public health recommendations [35], vaccine uptake [51, 52], and health outcomes [53] during the pandemic. In ongoing research, we are developing new strategies for engaging with conservative individuals (as identified by social media activity) to broaden our target audience and enhance the efficacy of our risk assessment tools.

A feature of our study is that we collected data prior to and during the Omicron wave in the United States, which encompassed several major holidays (Thanksgiving Day, Christmas, and New Year’s Eve). Overall, we found that our risk communication strategies remained effective throughout the 4-month period of data collection (S3 Fig). We explored temporal trends and found that participants were more likely to overestimate risk during November and December 2021, coinciding with the holiday season and the emergence of the Omicron variant. During the Omicron wave, participants tended to report greater decreases in willingness (S3 Fig). User engagement increased around holidays and the rise of the Omicron variant (S4 Fig). Our temporal analyses demonstrate that public perception of risk can fluctuate depending on disease prevalence, media attention, and holidays. Understanding these dynamics is crucial for epidemiological modeling of disease transmission and human behavior. Prior studies have demonstrated that public awareness and behavior change crucially shape the trajectory of pandemics [54–56]; incorporating human behavior into epidemiological models is essential for predicting the temporal dynamics of epidemics and the effects of public health policies [57, 58].

### Limitations

This dataset was collected via interactive tools on a public website with a large user base. This approach enabled large-scale online implementation of a prior evidence-based intervention, but necessarily included several limitations. Our sample was subject to self-selection bias; participants voluntarily sought out information about COVID-19 risk. Therefore, individuals who visited our website may be more concerned about COVID or interested in learning about exposure risk, relative to others who did not visit our website. However, we observed substantial risk misestimation in this self-selected sample, demonstrating that correcting risk perception was still necessary and beneficial in this population. In a related study [59], we reduced self-selection bias and expanded our reach by conducting large-scale advertising campaigns on social media. These advertising campaigns directed specific messages at different demographic groups; ads redirected social media users to the Event Risk Tool website, then tracked subsequent activity. Importantly, in this related study, we found that our interactive risk assessment tools were effective for both politically-liberal and conservative participants. Overall, selection bias limits our ability to generalize our findings to individuals who would not voluntarily seek information about COVID-19 risks. However, we demonstrate that intervention to correct risk misestimation is both necessary and beneficial for individuals who are willing to engage with risk information.

Relatedly, response biases are also possible; participants may be motivated to respond in a socially desirable way, reporting decreases in willingness to take risks. The pressure to respond in a socially-desirable way is likely lower in the present study relative to other psychology studies that use self-report measures, because the present study collected data from anonymous website visitors who did not receive payment or interact with an experimenter. However, it is impossible to eliminate socially-desirable responding in self-report measures. As a result of this response bias, the effects of the risk map and risk quiz decreasing willingness to take risks may be exaggerated. However, we expect that any effects of socially-desirable responding would consistently decrease reported willingness to take risks, regardless of other variables. We demonstrated that participants reported greater decreases in willingness for larger event sizes (after viewing the risk map) or after receiving feedback about risk underestimation (after completing the risk quiz). Our temporal analyses also demonstrated that changes in willingness fluctuated over the course of the omicron wave. Overall, although socially-desirable responding may contribute to some self-reported decreases in willingness to take risks, it is unlikely that this bias can account for the effects of event size and risk estimation feedback on risk tolerance. Future studies that measure real-world behavior could offer alternative measures of intervention outcomes that are not subject to response bias.

Additionally, for all analyses we identified users by public IP addresses, which are imperfect measures of unique identities; some individuals may share the same IP address, and others may change or hide their IP address. If an individual accessed the website via different devices connected to the same internet router (e.g., the same household), these visits would be recorded as multiple visits from the same user. However, if the same user accessed the website from multiple locations, or via a VPN, their activity would be recorded as if multiple unique users had accessed the website. Our estimated number of unique users may thus be greater than reality. Furthermore, some risk quiz data may have been erroneously excluded (because we analyzed only the first risk quiz submission from each unique user; see S1 Text). Note that due to the limitations of location tracking via IP addresses, we used user-verified location data to provide risk quiz feedback and investigate political differences across counties. Another related limitation is that we were not able to obtain subject-level metrics of general website activity (e.g., number of clicks or time spent on the map homepage); these usage statistics were measured by Google Analytics, but individual observations were not labelled by IP address and so cannot be integrated with survey or quiz responses.

Our risk assessment tools focus on event size, because the number of people that one encounters is the foundation of exposure risk. Calculating risk on the basis of event sizes allows us to efficiently communicate contextualized risk information, visualize risk on national and global maps, and transparently link risk estimates to COVID cases. However, the risk of *exposure* is distinct from the risk of *infection*. As stated on the website, other factors (e.g., vaccination, face coverings, ventilation, crowd density, and indoor vs. outdoor interactions) influence the risk of infection, and likely also an individual’s willingness to participate in an event. Other risk assessment tools attempt to account for additional factors that influence the risk of exposure and infection, prioritizing precision over accessibility [60]. Although such detailed tools are useful for individuals with adequate background knowledge, we argue that it is equally important to offer accessible risk communication tools that are easy to use and understand. Another limitation of our event-size analysis is that we did not assess risk estimation for the smallest event sizes (fewer than 20 people); data from the risk map indicated that participants were particularly interested in learning about the risk of small gatherings. Our results suggest that individuals may overestimate risk for events with fewer than 20 people (and so be more willing to engage in these activities after learning about prevalence-based exposure risk), but we did not test this directly in the present study.

Our prevalence estimates are also subject to several limitations. Population immunity, undertesting, inconsistencies and delays in case reporting, and infection rate can differ across regions. We used a default ascertainment bias value of 4, which is consistent with current CDC estimates, but this value may not be appropriate for all regions or all time periods. Our prevalence estimates also assume that some infectious individuals are engaging in public activities, but some individuals may isolate and thus reduce exposure risk.

There are also individual differences in risk perception and behavior. We used example scenarios for different event sizes (e.g., a coffee shop, a grocery store, or a graduation ceremony) to help participants visualize the scale of events and consider risk in daily life. Different participants may envision these scenarios in different ways. The scenarios we chose for larger event sizes were more ambiguous (e.g., a graduation ceremony or sporting event could take place indoors or outdoors). To verify that this ambiguity did not explain why risk misestimation differed across event sizes, we demonstrated that variance in risk estimation did not differ across event sizes (S4 Text). Lastly, we measured self-reported willingness to participate in events because we were unable to track real-world risky behavior; some participants may be more likely than others to act according to their stated intentions.

### Conclusion

The present study showcases the public health benefits of providing interactive tools for local, real-time risk assessment. Our data offer key insights for risk communication: Risk information can be made more engaging, memorable, and influential when it is geolocalized, interactive, and combines risk statistics with specific contextualizing scenarios (e.g., “Can you guess the current risk level for a wedding with 100 guests in your area?”). We argue that public health messaging may be most impactful if it emphasizes the risk of moderately large events (ranging from 25 to 1,000 people). Our participants were most likely to underestimate the risk of larger events, but viewing risk information substantially decreased willingness to participate in these events. Even within a self-selected sample of COVID-aware participants, we observed substantial risk misestimation, with a slight overall tendency towards underestimation of risk (especially for large event sizes). We propose that targeted messaging could help counteract pandemic fatigue, reduce viral transmission, inform public policy, and aid individual decision making. Beyond the COVID-19 pandemic, insights from our study can improve viral risk communication for other public health concerns such as monkeypox and influenza. These real-time risk assessment tools provide context for improved understanding of human behavior and risk misestimation that can be incorporated into epidemiological modeling underlying pandemic management and prevention.

## Supporting information

S1 Fig Exposure risk by group size. The risk that one or more people in a group are infectious (R) rises non-linearly with group size (n) for a given infectious prevalence (*p*) as R = 100×(1 - (1-*p*)^n^). Our approach to calculating *p* is described in detail in a prior report (Chande et al., 2020). When prevalence is high, even small events can be risky.
(DOCX)

S2 Fig Geographical distribution of users. Approximated geographical distribution of willingness rating submissions for the map homepage (A) and risk quizzes (B). Location data is inferred from IP addresses; these estimates are imperfect and we were not able to estimate location information for all users. These maps depict the approximate distribution of user engagement across the U.S.
(DOCX)

S3 Fig Temporal analysis of risk quiz data. Comparison of behavioral effects prior to the emergence of the omicron variant in the U.S. (before 12/01/2021; map willingness *n* = 4,473, risk quiz *n* = 2,707), during the early phase of the omicron wave (between 12/01/2021 and 12/31/2021; map willingness *n* = 1,622, risk quiz *n* = 953), and during the peak phase of the omicron wave (after 01/01/2022; map willingness *n* = 6,853, risk quiz *n* = 4,841). Overall, effects were consistent throughout the period of data collection. During the omicron wave, participants reported slightly greater decreases in willingness to participate in moderately-large events (panels A, B, and C). During the omicron wave, participants were somewhat more accurate at estimating risk, although there was still substantial underestimation of risk for large events (panels D, E, and F). During the peak of the omicron wave, Underestimators reported greater decreases in willingness after completing the risk quiz (panels G, H, and I).
(DOCX)

S4 Fig User engagement with the website over time. We observed a very large increase in user engagement around the Thanksgiving holiday (which coincided with some holiday-related press coverage), and a smaller peak around Christmas. Note that the Thanksgiving peak coincided with significant press coverage about the website. Baseline user engagement increased during the omicron wave. A) Histogram of willingness ratings submitted on the map homepage over time. B) Histogram of risk quiz submissions over time. C) Histogram of website press coverage (online and televised news sources) during the data collection period.
(DOCX)

S1 Table Descriptive statistics for risk map data. Table of descriptive statistics and one-sample t-statistics for the average *change in willingness* reported after viewing the U.S. risk map for each event size. Not all participants viewed and submitted a willingness rating for all possible event sizes, resulting in different sample sizes for each event size. The default event size (first shown when the website loads) was 50 people. One-sample t-tests were corrected for multiple comparisons with Tukey’s HSD.
(DOCX)

S2 Table Descriptive statistics for risk quiz data. A) Descriptive statistics and one-sample t-statistics for the average *risk estimation error* across event sizes. B) Statistical tests for pairwise comparisons of average risk estimation error among the four event sizes (20, 50, 100, and 1000 people) assessed by the risk quiz. Participants overestimated the risk of small events (20 people), but underestimated the risk of large events (100 and 1000 people). One-sample and pairwise tests were corrected for multiple comparisons with Tukey’s HSD.
(DOCX)

S3 Table Advertisement campaigns conducted on social media during the data collection period. Several small-scale advertising campaigns were conducted on Facebook and Instagram in early November 2021, followed by several larger-scale, holiday-themed advertising campaigns. The purpose of these ads was to direct traffic to the Event Risk Tool website. Most of these ads broadly targeted a general audience on Facebook or Instagram (all users aged 18+ years, currently residing in the United States). Two small campaigns targeted Facebook users identified as politically conservative, as determined by Facebook’s classification of the user’s activity on the platform.
(DOCX)

S4 Table Analysis of risk estimation error by political leaning. Parameter estimates from a linear mixed effects regression model predicting *Risk Estimation Error* (averaged across event sizes) from the county-level variables *Conservative Vote* (% vote for the Republican party in the 2020 presidential election), *COVID-19 Cases* (number of active cases per 100,000 people), and *Total Voters* (for the 2020 presidential election). The model included random intercepts for US counties. Degrees of freedom were estimated with Sattherthwaite’s method.
(DOCX)

S5 Table Analysis predicting post-quiz change in willingness from political leaning. Parameter estimates from a linear mixed effects regression model predicting *Change in Willingness* after the risk quiz from the variables *Risk Estimation Error* (averaged across event sizes), *Conservative Vote* (% vote for the Republican party in the 2020 presidential election), the interaction between *Risk Estimation Error* and *Conservative Vote*, *COVID-19 Cases* (number of active cases per 100,000 people), and *Total Voters* (for the 2020 presidential election). The model included random intercepts for US counties. Degrees of freedom were estimated with Sattherthwaite’s method.
(DOCX)

S1 Text Supporting methodological information. (DOCX)

S2 Text Local mask-wearing policies. (DOCX)

S3 Text Qualtrics demographics survey. (DOCX)

S4 Text Variance in risk estimation. (DOCX)

S1 File (PDF)

S2 File (PDF)
