# Supplementary material for: Communicating COVID-19 exposure risk with an interactive website counteracts risk misestimation
Source: PLoS One. 2023 Oct 5;18(10):e0290708. doi: 10.1371/journal.pone.0290708 (PMC10553796; doi:10.1371/journal.pone.0290708)
Supplement: S1 Fig — The risk that one or more people in a group are infectious (R) rises non-linearly with group size (n) for a given infectious prevalence (p) as R = 100×(1 - (1-p)n). Our approach to calculating p is described in detail in a prior report (Chande et al., 2020). When prevalence is high, even small events can be risky. (DOCX) [file pone.0290708.s001.docx]

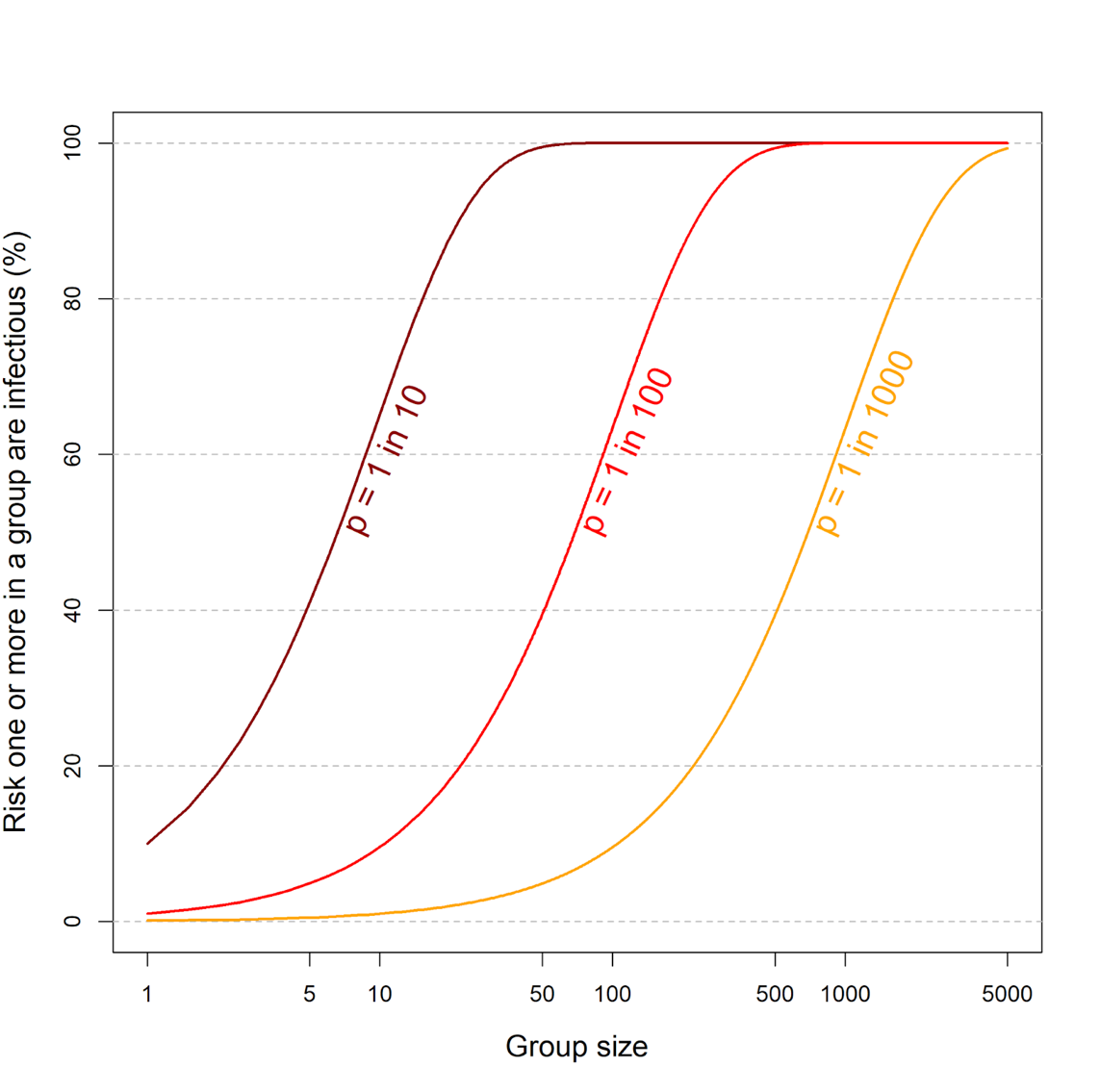

**S1 Figure**. **Exposure risk by group size.** The risk that one or more people in a group are infectious (R) rises non-linearly with group size (n) for a given infectious prevalence (*p*) as R = 100×(1 - (1-*p*)^n^). Our approach to calculating *p* is described in detail in a prior report (Chande et al., 2020). When prevalence is high, even small events can be risky.
