# Supplementary material for: Communicating COVID-19 exposure risk with an interactive website counteracts risk misestimation
Source: PLoS One. 2023 Oct 5;18(10):e0290708. doi: 10.1371/journal.pone.0290708 (PMC10553796; doi:10.1371/journal.pone.0290708)
Supplement: S2 Fig — Approximated geographical distribution of willingness rating submissions for the map homepage (A) and risk quizzes (B). Location data is inferred from IP addresses; these estimates are imperfect and we were not able to estimate location information for all users. These maps depict the approximate distribution of user engagement across the U.S. (DOCX) [file pone.0290708.s002.docx]

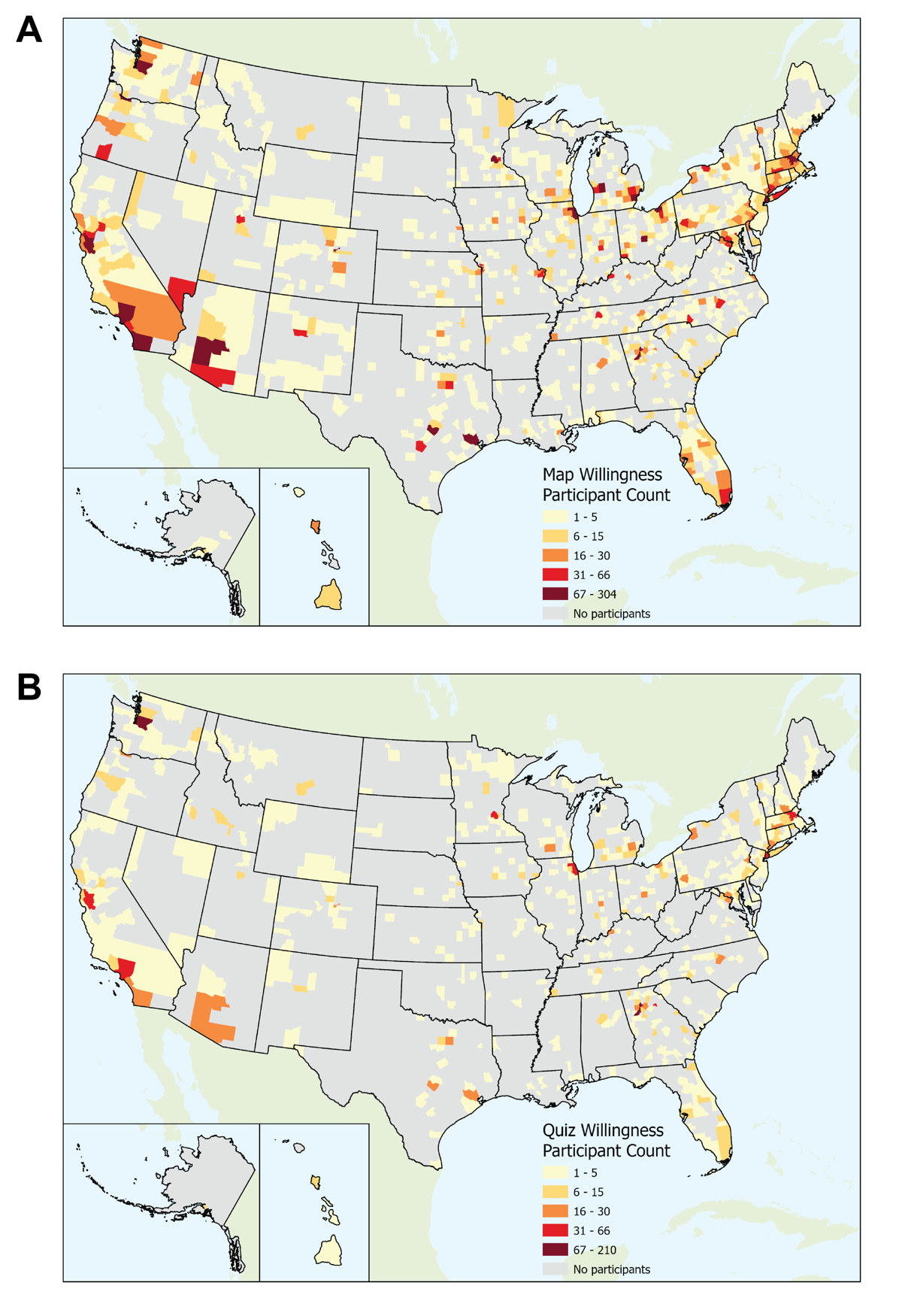


**S2 Figure***.* **Geographical distribution of users.** Approximated geographical distribution of willingness rating submissions for the map homepage (A) and risk quizzes (B). Location data is inferred from IP addresses; these estimates are imperfect and we were not able to estimate location information for all users. These maps depict the approximate distribution of user engagement across the U.S.
