# Supplementary material for: Communicating COVID-19 exposure risk with an interactive website counteracts risk misestimation
Source: PLoS One. 2023 Oct 5;18(10):e0290708. doi: 10.1371/journal.pone.0290708 (PMC10553796; doi:10.1371/journal.pone.0290708)
Supplement: S3 Fig — Comparison of behavioral effects prior to the emergence of the omicron variant in the U.S. (before 12/01/2021; map willingness n = 4,473, risk quiz n = 2,707), during the early phase of the omicron wave (between 12/01/2021 and 12/31/2021; map willingness n = 1,622, risk quiz n = 953), and during the peak phase of the omicron wave (after 01/01/2022; map willingness n = 6,853, risk quiz n = 4,841). Overall, effects were consistent throughout the period of data collection. During the omicron wave, participants reported slightly greater decreases in willingness to participate in moderately-large events (panels A, B, and C). During the omicron wave, participants were somewhat more accurate at estimating risk, although there was still substantial underestimation of risk for large events (panels D, E, and F). During the peak of the omicron wave, Underestimators reported greater decreases in willingness after completing the risk quiz (panels G, H, and I). (DOCX) [file pone.0290708.s003.docx]

**
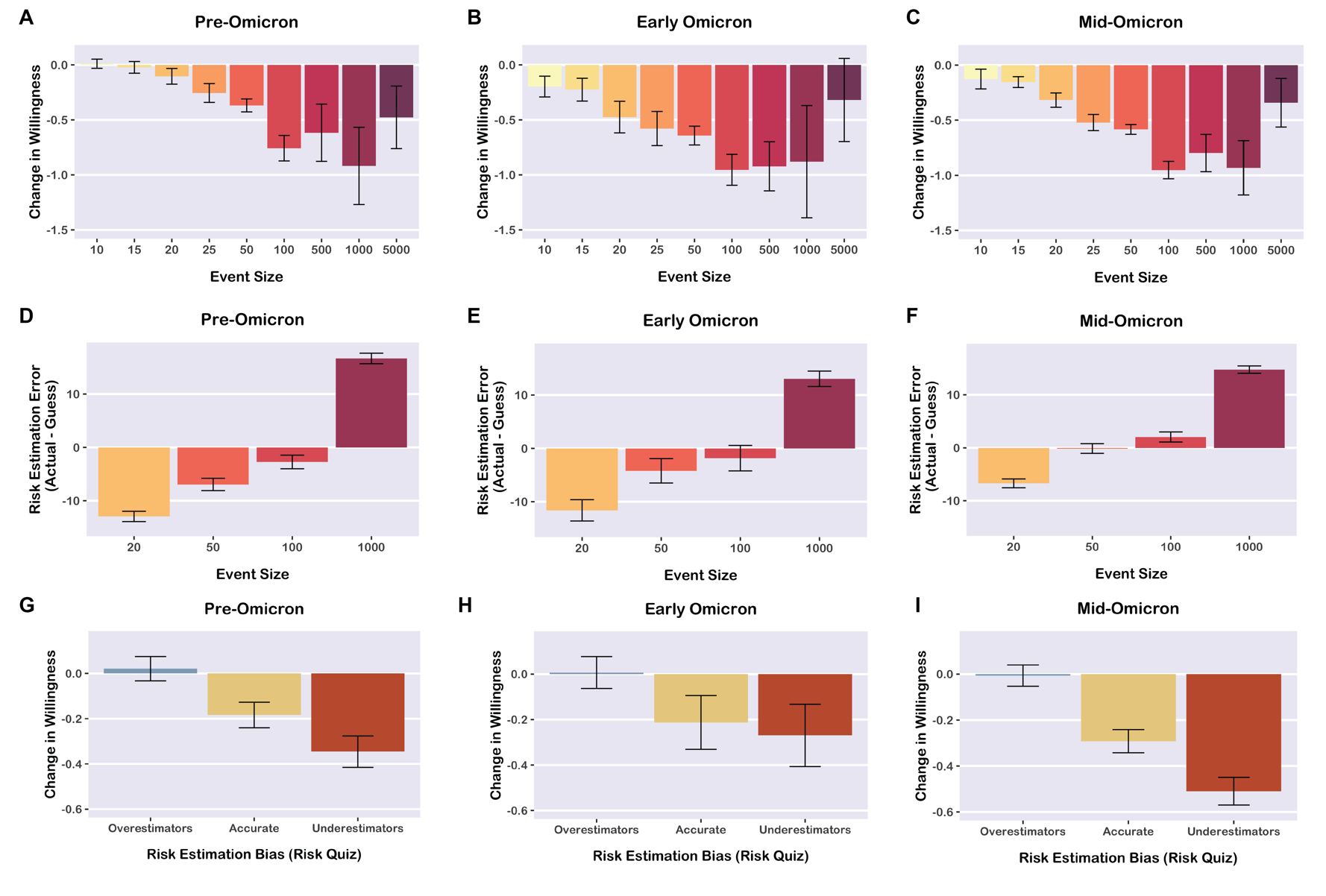
S3 Figure.** **Temporal analysis of risk quiz data**. Comparison of behavioral effects prior to the emergence of the omicron variant in the U.S. (before 12/01/2021; map willingness *n* = 4,473, risk quiz *n* = 2,707), during the early phase of the omicron wave (between 12/01/2021 and 12/31/2021; map willingness *n* = 1,622, risk quiz *n* = 953), and during the peak phase of the omicron wave (after 01/01/2022; map willingness *n* = 6,853, risk quiz *n* = 4,841). Overall, effects were consistent throughout the period of data collection. During the omicron wave, participants reported slightly greater decreases in willingness to participate in moderately-large events (panels A, B, and C). During the omicron wave, participants were somewhat more accurate at estimating risk, although there was still substantial underestimation of risk for large events (panels D, E, and F). During the peak of the omicron wave, Underestimators reported greater decreases in willingness after completing the risk quiz (panels G, H, and I).
