# Supplementary material for: Communicating COVID-19 exposure risk with an interactive website counteracts risk misestimation
Source: PLoS One. 2023 Oct 5;18(10):e0290708. doi: 10.1371/journal.pone.0290708 (PMC10553796; doi:10.1371/journal.pone.0290708)
Supplement: S4 Fig — We observed a very large increase in user engagement around the Thanksgiving holiday (which coincided with some holiday-related press coverage), and a smaller peak around Christmas. Note that the Thanksgiving peak coincided with significant press coverage about the website. Baseline user engagement increased during the omicron wave. A) Histogram of willingness ratings submitted on the map homepage over time. B) Histogram of risk quiz submissions over time. C) Histogram of website press coverage (online and televised news sources) during the data collection period. (DOCX) [file pone.0290708.s004.docx]

**
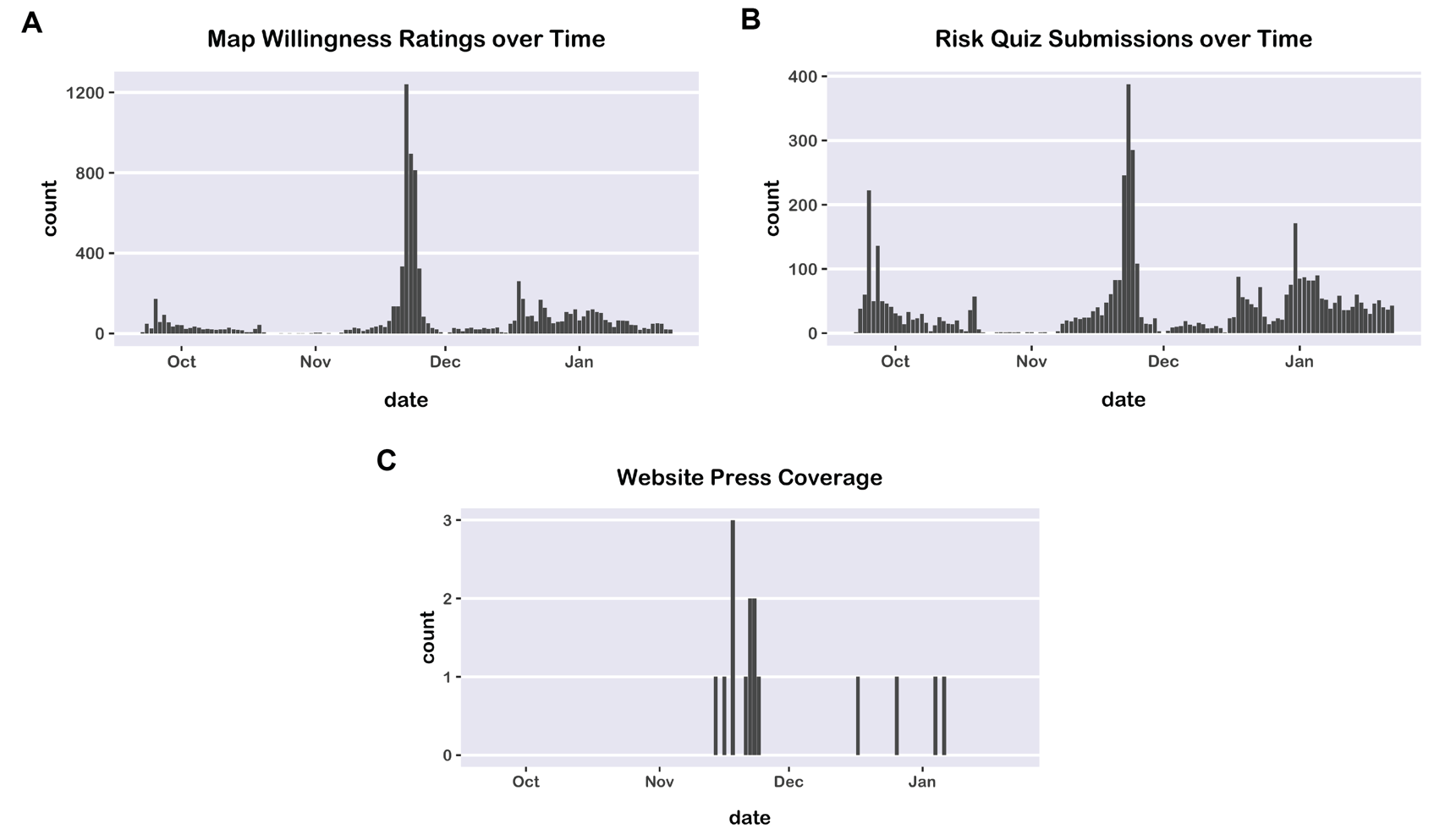
S4 Figure.** **User engagement with the website over time.** We observed a very large increase in user engagement around the Thanksgiving holiday (which coincided with some holiday-related press coverage), and a smaller peak around Christmas. Note that the Thanksgiving peak coincided with significant press coverage about the website. Baseline user engagement increased during the omicron wave. A) Histogram of willingness ratings submitted on the map homepage over time. B) Histogram of risk quiz submissions over time. C) Histogram of website press coverage (online and televised news sources) during the data collection period.
