# Supplementary material for: Communicating COVID-19 exposure risk with an interactive website counteracts risk misestimation
Source: PLoS One. 2023 Oct 5;18(10):e0290708. doi: 10.1371/journal.pone.0290708 (PMC10553796; doi:10.1371/journal.pone.0290708)
Supplement: S1 Table — Table of descriptive statistics and one-sample t-statistics for the average change in willingness reported after viewing the U.S. risk map for each event size. Not all participants viewed and submitted a willingness rating for all possible event sizes, resulting in different sample sizes for each event size. The default event size (first shown when the website loads) was 50 people. One-sample t-tests were corrected for multiple comparisons with Tukey’s HSD. (DOCX) [file pone.0290708.s005.docx]

**S1 Table.** **Descriptive statistics for risk map data.** Table of descriptive statistics and one-sample t-statistics for the average *change in willingness* reported after viewing the U.S. risk map for each event size. Not all participants viewed and submitted a willingness rating for all possible event sizes, resulting in different sample sizes for each event size. The default event size (first shown when the website loads) was 50 people. One-sample t-tests were corrected for multiple comparisons with Tukey’s HSD.

| **Event Size** | **N** | **Mean** | **SD** | **T-Statistic** | **P-Value** |
| --- | --- | --- | --- | --- | --- |
| 10 people | 2433 | -0.17 | 0.99 | -8.14 | < 0.0001 |
| 15 people | 1233 | -0.15 | 0.88 | -5.33 | < 0.0001 |
| 20 people | 852 | -0.32 | 0.97 | -9.14 | < 0.0001 |
| 25 people | 690 | -0.52 | 0.98 | -13.49 | < 0.0001 |
| 50 people | 2258 | -0.56 | 1.08 | -27.14 | < 0.0001 |
| 100 people | 664 | -0.95 | 1.04 | -24.14 | < 0.0001 |
| 500 people | 203 | -0.80 | 1.22 | -11.19 | < 0.0001 |
| 1,000 people | 89 | -0.93 | 1.17 | -8.66 | < 0.0001 |
| 5,000 people | 155 | -0.34 | 1.39 | -4.19 | 0.0003 |
