# Supplementary material for: Communicating COVID-19 exposure risk with an interactive website counteracts risk misestimation
Source: PLoS One. 2023 Oct 5;18(10):e0290708. doi: 10.1371/journal.pone.0290708 (PMC10553796; doi:10.1371/journal.pone.0290708)
Supplement: S2 Table — A) Descriptive statistics and one-sample t-statistics for the average risk estimation error across event sizes. B) Statistical tests for pairwise comparisons of average risk estimation error among the four event sizes (20, 50, 100, and 1000 people) assessed by the risk quiz. Participants overestimated the risk of small events (20 people), but underestimated the risk of large events (100 and 1000 people). One-sample and pairwise tests were corrected for multiple comparisons with Tukey’s HSD. (DOCX) [file pone.0290708.s006.docx]

**S2 Table***.* **Descriptive statistics for risk quiz data.** A) Descriptive statistics and one-sample t-statistics for the average *risk estimation error* across event sizes. B) Statistical tests for pairwise comparisons of average risk estimation error among the four event sizes (20, 50, 100, and 1000 people) assessed by the risk quiz. Participants overestimated the risk of small events (20 people), but underestimated the risk of large events (100 and 1000 people). One-sample and pairwise tests were corrected for multiple comparisons with Tukey’s HSD.

| **S2A: Descriptive statistics for risk estimation error** | | | | | |
| --- | --- | --- | --- | --- | --- |
| **Event Size** | **N** | **Mean** | **SD** | **Z-Statistic** | **P-Value** |
| 20 people | 4841 | 6.72 | 30.23 | 15.30 | < 0.0001 |
| 50 people | 4841 | 0.14 | 33.11 | 0.32 | 0.751 |
| 100 people | 4841 | -2.05 | 33.54 | -4.66 | < 0.0001 |
| 1000 people | 4841 | -14.77 | 24.54 | -33.62 | < 0.0001 |

| **S2B: Pairwise contrasts for risk estimation error** | | | |
| --- | --- | --- | --- |
| **Contrast (Event Size)** | **Parameter Estimate** | **Z-Statistic** | **P-Value** |
| 20 people > 50 people | 6.58 | 17.34 | < 0.0001 |
| 20 people > 100 people | 8.77 | 23.10 | < 0.0001 |
| 20 people > 1000 people | 21.49 | 56.62 | < 0.0001 |
| 50 people > 100 people | 2.18 | 5.76 | < 0.0001 |
| 50 people > 1000 people | 14.91 | 39.28 | < 0.0001 |
| 100 people > 1000 people | 12.72 | 33.52 | < 0.0001 |
