# Supplementary material for: Communicating COVID-19 exposure risk with an interactive website counteracts risk misestimation
Source: PLoS One. 2023 Oct 5;18(10):e0290708. doi: 10.1371/journal.pone.0290708 (PMC10553796; doi:10.1371/journal.pone.0290708)
Supplement: S3 Table — Several small-scale advertising campaigns were conducted on Facebook and Instagram in early November 2021, followed by several larger-scale, holiday-themed advertising campaigns. The purpose of these ads was to direct traffic to the Event Risk Tool website. Most of these ads broadly targeted a general audience on Facebook or Instagram (all users aged 18+ years, currently residing in the United States). Two small campaigns targeted Facebook users identified as politically conservative, as determined by Facebook’s classification of the user’s activity on the platform. (DOCX) [file pone.0290708.s007.docx]

**S3 Table.** **Advertisement campaigns conducted on social media during the data collection period.** Several small-scale advertising campaigns were conducted on Facebook and Instagram in early November 2021, followed by several larger-scale, holiday-themed advertising campaigns. The purpose of these ads was to direct traffic to the Event Risk Tool website. Most of these ads broadly targeted a general audience on Facebook or Instagram (all users aged 18+ years, currently residing in the United States). Two small campaigns targeted Facebook users identified as politically conservative, as determined by Facebook’s classification of the user’s activity on the platform.

| **Campaign** | **Platform** | **Audience** | **Clicks** | **Click Rate** | **Dates** |
| --- | --- | --- | --- | --- | --- |
| Quiz emphasis | Facebook | Conservatives | 141 | 8.2% | 11/8/21 - 11/15/21 |
| Local risk emphasis | Facebook | Conservatives | 327 | 9.5% | 11/8/21 - 11/15/21 |
| Risk map image, with text | Instagram | General | 130 | 2.9% | 11/8/21 - 11/15/21 |
| Thanksgiving | Instagram | General | 152 | 3.2% | 11/8/21 - 11/15/21 |
| Thanksgiving | Facebook | General | 1330 | 9.7% | 11/19/21 - 11/28/21 |
| Christmas | Facebook | General | 992 | 8.6% | 12/12/21 - 12/26/21 |
| New Year's Eve | Facebook | General | 591 | 7.5% | 12/24/21 - 1/1/22 |
