# Supplementary material for: Communicating COVID-19 exposure risk with an interactive website counteracts risk misestimation
Source: PLoS One. 2023 Oct 5;18(10):e0290708. doi: 10.1371/journal.pone.0290708 (PMC10553796; doi:10.1371/journal.pone.0290708)
Supplement: S4 Table — Parameter estimates from a linear mixed effects regression model predicting Risk Estimation Error (averaged across event sizes) from the county-level variables Conservative Vote (% vote for the Republican party in the 2020 presidential election), COVID-19 Cases (number of active cases per 100,000 people), and Total Voters (for the 2020 presidential election). The model included random intercepts for US counties. Degrees of freedom were estimated with Sattherthwaite’s method. (DOCX) [file pone.0290708.s008.docx]

**S4 Table.** **Analysis of risk estimation error by political leaning**. Parameter estimates from a linear mixed effects regression model predicting *Risk Estimation Error* (averaged across event sizes) from the county-level variables *Conservative Vote* (% vote for the Republican party in the 2020 presidential election), *COVID-19 Cases* (number of active cases per 100,000 people), and *Total Voters* (for the 2020 presidential election). The model included random intercepts for US counties. Degrees of freedom were estimated with Sattherthwaite’s method.

|  |  | **Dependent Variable: Risk Estimation Error** | | | |
| --- | --- | --- | --- | --- | --- |
| *Predictors* | *Estimates* | *CI* | *t* | *p* | *df* |
| (Intercept) | 0.00 | -0.04 – 0.04 | 0.11 | 0.912 | 152 |
| Conservative Vote | -0.03 | -0.07 – 0.01 | -1.52 | 0.130 | 485 |
| COVID-19 Cases | -0.33 ^***^ | -0.36 – -0.30 | -20.74 | **<0.001** | 3642 |
| Total Voters | 0.02 | -0.03 – 0.07 | 0.87 | 0.386 | 71 |
| Observations |  | 3837 | | | |
| Marginal R^2^ / Conditional R^2^ |  | 0.105 / 0.140 | | | |
|  | ** p<0.05   ** p<0.01   *** p<0.001* | | | | |
