# Supplementary material for: Communicating COVID-19 exposure risk with an interactive website counteracts risk misestimation
Source: PLoS One. 2023 Oct 5;18(10):e0290708. doi: 10.1371/journal.pone.0290708 (PMC10553796; doi:10.1371/journal.pone.0290708)
Supplement: S5 Table — Parameter estimates from a linear mixed effects regression model predicting Change in Willingness after the risk quiz from the variables Risk Estimation Error (averaged across event sizes), Conservative Vote (% vote for the Republican party in the 2020 presidential election), the interaction between Risk Estimation Error and Conservative Vote, COVID-19 Cases (number of active cases per 100,000 people), and Total Voters (for the 2020 presidential election). The model included random intercepts for US counties. Degrees of freedom were estimated with Sattherthwaite’s method. (DOCX) [file pone.0290708.s009.docx]

**S5 Table.** **Analysis predicting post-quiz change in willingness from political leaning**. Parameter estimates from a linear mixed effects regression model predicting *Change in Willingness* after the risk quiz from the variables *Risk Estimation Error* (averaged across event sizes), *Conservative Vote* (% vote for the Republican party in the 2020 presidential election), the interaction between *Risk Estimation Error* and *Conservative Vote*, *COVID-19 Cases* (number of active cases per 100,000 people), and *Total Voters* (for the 2020 presidential election). The model included random intercepts for US counties. Degrees of freedom were estimated with Sattherthwaite’s method.

|  |  | **Dependent Variable: Post-Quiz Change in Willingness** | | | |
| --- | --- | --- | --- | --- | --- |
| *Predictors* | *Estimates* | *CI* | *t* | *p* | *df* |
| (Intercept) | -0.06 ^***^ | -0.11 – -0.02 | -2.74 | **0.006** | 163 |
| Risk Estimation Error | 0.17 ^***^ | 0.13 – 0.22 | 7.14 | **<0.001** | 1822 |
| Conservative Vote | 0.05 | 0.00 – 0.10 | 1.96 | 0.050 | 265 |
| COVID-19 Cases | -0.32 ^***^ | -0.37 – -0.27 | -12.78 | **<0.001** | 1779 |
| Total Voters | 0.02 | -0.03 – 0.07 | 0.69 | 0.490 | 50 |
| Risk Estimation Error *  Conservative Vote | -0.10 ^***^ | -0.14 – -0.05 | -4.01 | **<0.001** | 1813 |
| Observations |  | 1830 | | | |
| Marginal R^2^ / Conditional R^2^ |  | 0.143 / 0.148 | | | |
|  | ** p<0.05   ** p<0.01   *** p<0.001* | | | | |
