# Supplementary material for: Communicating COVID-19 exposure risk with an interactive website counteracts risk misestimation
Source: PLoS One. 2023 Oct 5;18(10):e0290708. doi: 10.1371/journal.pone.0290708 (PMC10553796; doi:10.1371/journal.pone.0290708)
Supplement: S1 Text — (DOCX) [file pone.0290708.s010.docx]

**S1 Text. Supporting Methodological Information**

***R Package Versions***

Data cleaning, analysis, and visualization utilized the R packages plyr^1^ (v1.8.6), dplyr^2^ (v1.0.7), tidyr^3^ (v1.1.4), ggplot2^4^ (v2.3.3.5), sjPlot^5^ (v2.8.9), lme4^6^ (v1.1-27.1), lmerTest^7^ (v3.1-3), car^8^ (v3.0-4), Rmisc^9^ (v1.5), emmeans^10^ (v1.7.0), effectsize^11^ (v0.5), lubridate^12^ (1.8.0), and stringr^13^ (1.4.0).

***Risk Quiz Exclusions***

Although the slight majority of users (56%) only submitted one risk quiz, others submitted multiple quizzes (ranging from 2 to 37 submissions). Some users may utilize the risk quiz repeatedly to check the risk levels for other locations, check if risk has changed over time, or try to improve their score. For the analyses reported in the main text, we opted to analyze only the first risk quiz submission from each user, excluding any subsequent submissions. This approach has two main benefits: 1) The first quiz offers insight into a user’s risk estimation bias prior to receiving accuracy feedback, and 2) Excluding multiple submissions from some users prevents a statistical issue of non-independent observations.

Additionally, some users (17%) submitted risk quizzes with all-default responses (50% for every event size). We excluded these default submissions from the analyses reported in the main text because they may indicate that the user submitted the quiz prematurely, did not wish to guess, or did not understand the tool. However, we also conducted the same analyses with all risk quiz submissions (including repeat and default submissions) to ensure that these exclusions did not meaningfully impact our results. We found that all of the results reported in the main text were still evident when we did not exclude any risk quiz submissions, as summarized below.

There was a small bias towards underestimating risk (mean risk estimation error = +4.31 points, *t*_(8967)_ = 16.51, *p <* 0*.*0001, Cohen’s *d* = 0.17, 95% CI [0.15, 0.20]). There was also a significant effect of event size on risk estimation error (*F*_(3,26029)_ = 2436.9, *p <* 0*.*0001). After viewing feedback from the risk quiz, participants reported a significant average decrease in willingness to participate in events (*t*_(3474)_ = -21.92, *p <* 0*.*0001, *d* = -0.37, 95% CI [-0.41, -0.34]). Lastly, there was an effect of risk estimation bias (*Underestimator, Accurate Estimator,* or *Overestimator*) on change in willingness after the risk quiz (*F*_(2,3472)_ = 78.93, *p <* 0*.*0001).

For the analysis of political partisanship, we excluded risk quiz data from 970 users (2%) who selected the default location rather than their own location. Additionally, the COVID-19 case data source compiled data from across all boroughs of New York City, whereas the political data source separated voting information by borough. Therefore, we averaged political data across New York City to enable integration with the COVID-19 data.

**References**

1. Wickham, H. The Split-Apply-Combine Strategy for Data Analysis. *Journal of Statistical Software* **40**, 1–29 (2011).

2. Wickham, H., François, R., Henry, L. & Müller, K. *dplyr: A Grammar of Data Manipulation*. (2021).

3. Wickham, H. *tidyr: Tidy Messy Data*. (2021).

4. Wickham, H. *ggplot2: Elegant Graphics for Data Analysis*. (Springer-Verlag New York, 2016).

5. Lüdecke, D. *sjPlot: Data Visualization for Statistics in Social Science*. (2021).

6. Bates, D., Mächler, M., Bolker, B. & Walker, S. Fitting linear mixed-effects models using lme4. (2014).

7. Kuznetsova, A., Brockhoff, P. B. & Christensen, R. H. B. lmerTest Package: Tests in Linear Mixed Effects Models. *Journal of Statistical Software* **82**, 1–26 (2017).

8. Fox, J. & Weisberg, S. *An R Companion to Applied Regression*. (Sage, 2019).

9. Hope, R. M. *Rmisc*. (2013).

10. Lenth, R. V. *emmeans: Estimated Marginal Means, aka Least-Squares Means*. (2021).

11. Ben-Shachar, M. S., Lüdecke, D. & Makowski, D. effectsize: Estimation of Effect Size Indices and Standardized Parameters. *Journal of Open Source Software* **5**, 2815 (2020).

12. Grolemund, G. & Wickham, H. Dates and Times Made Easy with lubridate. *Journal of Statistical Software* **40**, 1–25 (2011).

13. Wickham, H. *stringr: Simple, Consistent Wrappers for Common String Operations*. (2019).
