# Supplementary material for: Communicating COVID-19 exposure risk with an interactive website counteracts risk misestimation
Source: PLoS One. 2023 Oct 5;18(10):e0290708. doi: 10.1371/journal.pone.0290708 (PMC10553796; doi:10.1371/journal.pone.0290708)
Supplement: S2 Text — (DOCX) [file pone.0290708.s011.docx]

**S2 Text. Local Mask-Wearing Policies**

Local public health restrictions may differ systematically across liberal and conservative counties, and in turn influence individuals’ willingness to participate in public gatherings. We obtained data on COVID-19 policies from the COVID Analysis and Mapping of Policies Project, which maintains a database of pandemic-related policies (<https://covid-local.org/amp/>). Few strict restrictions (e.g., business closures or limitations on gatherings) were in effect at the time that the study was conducted. However, many U.S. counties enacted mask-wearing policies during the study period. These policies were heterogeneous, but either mandated or recommended masks in public settings. Therefore, we conducted a control analysis testing whether local mask-wearing policies influenced the effect of the risk quiz on change in willingness to participate in potentially-risky events (Table). For each participant, we determined whether any mask-wearing policy was in effect in the participant’s county, city, or state on the date that the participant visited the website; 61.4% of participants were in a location with a mask policy in effect.

**Table***.* **Analysis predicting change in willingness from local mask policies.** *|*Parameter estimates from a linear mixed effects regression model predicting *Change in Willingness* after the risk quiz from the variables *Risk Estimation Error* (averaged across event sizes), *Conservative Vote* (% vote for the Republican party in the 2020 presidential election), *Mask Policy* (True or False), *COVID-19 Cases* (number of active cases per 100,000 people), *Total Voters* (for the 2020 presidential election), and all interactions of interest. The model included random intercepts for US counties. Degrees of freedom were estimated with Sattherthwaite’s method.

|  | **Dependent Variable: Change in Willingness** | | | |
| --- | --- | --- | --- | --- |
| *Predictors* | *Estimates* | *CI* | *p* | *df* |
| (Intercept) | -0.06 ^**^ | -0.11 – -0.02 | **0.007** | 176 |
| Risk Estimation Error | 0.18 ^***^ | 0.13 – 0.23 | **<0.001** | 1819 |
| Conservative Vote | 0.05 | -0.00 – 0.10 | 0.076 | 297 |
| Mask Policy | -0.01 | -0.05 – 0.04 | 0.777 | 194 |
| COVID-19 Cases | -0.32 ^***^ | -0.37 – -0.27 | **<0.001** | 1777 |
| Total Voters | 0.02 | -0.04 – 0.07 | 0.517 | 47 |
| Risk Estimation Error * Conservative Vote | -0.09 ^***^ | -0.14 – -0.05 | **<0.001** | 1804 |
| Risk Estimation Error * Mask Policy | 0.02 | -0.02 – 0.07 | 0.309 | 1815 |
| Conservative Vote * Mask Policy | -0.01 | -0.05 – 0.04 | 0.802 | 196 |
| Risk Estimation Error * Conservative Vote  * Mask Policy | 0.01 | -0.04 – 0.05 | 0.775 | 1801 |
| Observations | 1830 | | | |
| Marginal R^2^ / Conditional R^2^ | 0.143 / 0.150 | | | |
| ** p<0.05   ** p<0.01   *** p<0.001* | | | | |
