# Supplementary material for: Communicating COVID-19 exposure risk with an interactive website counteracts risk misestimation
Source: PLoS One. 2023 Oct 5;18(10):e0290708. doi: 10.1371/journal.pone.0290708 (PMC10553796; doi:10.1371/journal.pone.0290708)
Supplement: S3 Text — (DOCX) [file pone.0290708.s012.docx]

**S3 Text. Qualtrics Demographics Survey**

Figures A-D were generated from data obtained with a demographics survey hosted by Qualtrics (*N* = 612). Survey responses were collected between 11/17/21 and 1/22/22. Using a banner displayed on the website, we invited visitors to complete the optional survey (approximately 2 minutes in duration) for a chance to win a $50 Amazon gift card in a monthly lottery draw. The plots below offer some insight into our user base, but they should be interpreted with caution because of self-selection bias (i.e., only a small subset of website visitors elect to complete the survey). The majority of our survey respondents were female, middle-aged, politically liberal, and vaccinated against COVID-19.

Exploratory analyses did not reveal any significant effects of age, gender, or political leaning on either risk estimation accuracy or post-quiz change in willingness to participate in events (Table). The Qualtrics survey sample did not provide sufficient statistical powered to detect such effects; these exploratory analyses depended on the small subset of data from participants who 1) used the interactive website tools, 2) completed the Qualtrics survey, and 3) had a valid IP address to link data from multiple sources.

**Table***.* Parameter estimates from linear regression models predicting risk estimation error (A) and post-quiz change in willingness (B) from demographic variables measured in the Qualtrics survey: political attitudes (Liberal vs. Conservative/Moderate), age (continuous variable), and gender.

|  | **A) Dependent Variable: Risk Estimation Error** | | | |
| --- | --- | --- | --- | --- |
| *Predictors* | *Estimates* | *CI* | *p* | *df* |
| (Intercept) | -0.31 ^***^ | -0.44 – -0.18 | **<0.001** | 263.00 |
| Political Attitudes | -0.11 | -0.24 – 0.02 | 0.087 | 263.00 |
| Age | -0.08 | -0.26 – 0.11 | 0.411 | 263.00 |
| Gender | 0.09 | -0.03 – 0.22 | 0.148 | 263.00 |
|  | **B) Dependent Variable: Change in Willingness** | | | |
| *Predictors* | *Estimates* | *CI* | *p* | *df* |
| (Intercept) | 0.18 ^*^ | 0.04 – 0.32 | **0.014** | 188.00 |
| Political Attitudes | 0.07 | -0.07 – 0.20 | 0.340 | 188.00 |
| Age | -0.00 | -0.21 – 0.20 | 0.962 | 188.00 |
| Gender | 0.04 | -0.10 – 0.17 | 0.580 | 188.00 |
| ** p<0.05   ** p<0.01   *** p<0.001* | | | | |


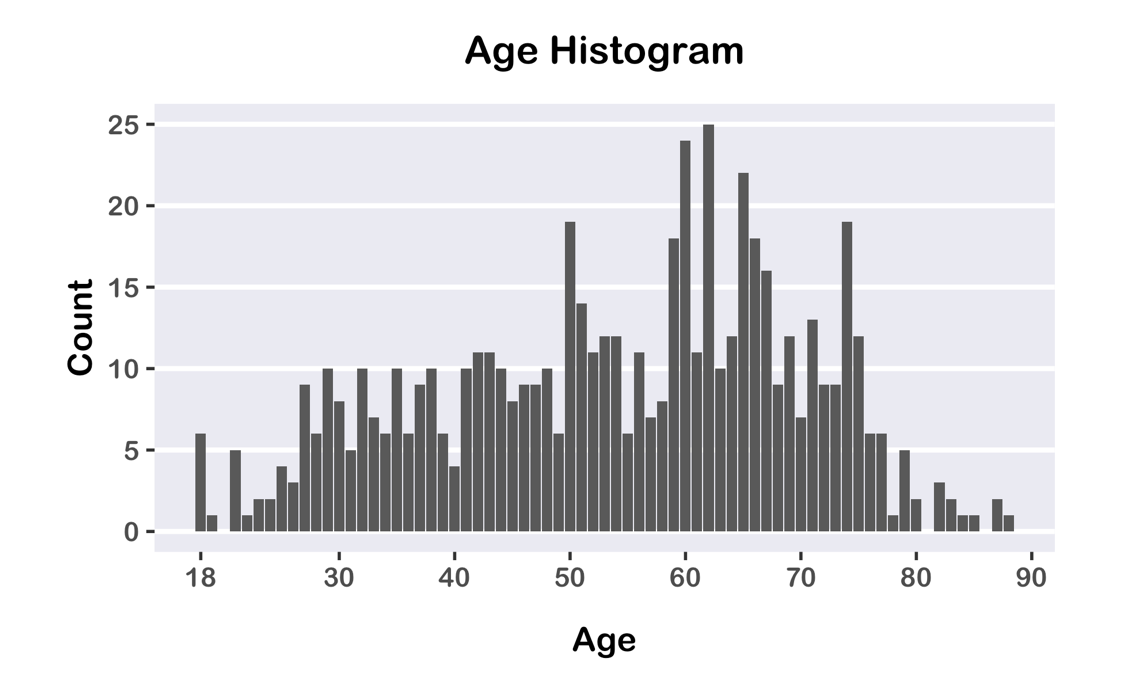


*Figure A*. Histogram of age (in years) reported by survey respondents. Participants ranged in age from 18 to 88. Note that participants who reported being under the age of 18 were screened out of the survey.


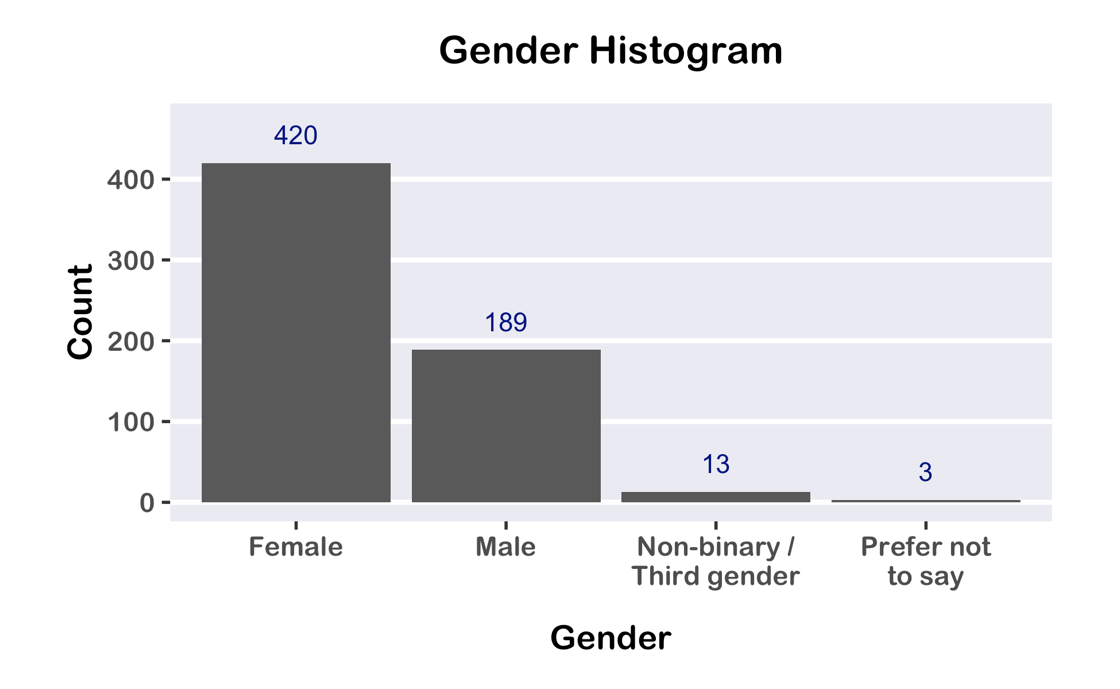

*Figure B*. Histogram of gender reported by survey respondents. The majority of survey respondents were female. Blue numbers above bars indicate counts.


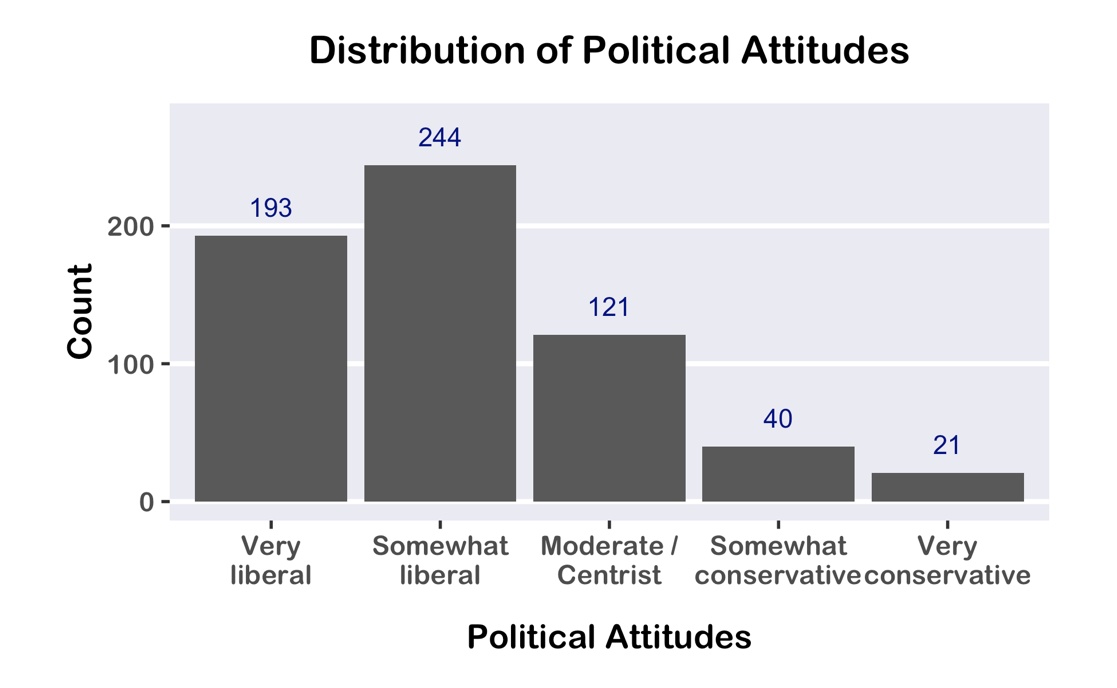

*Figure C.* Histogram of political attitudes reported by survey respondents. The majority of participants were liberal-leaning, but 30% identified as moderate or conservative. Blue numbers above bars indicate counts.


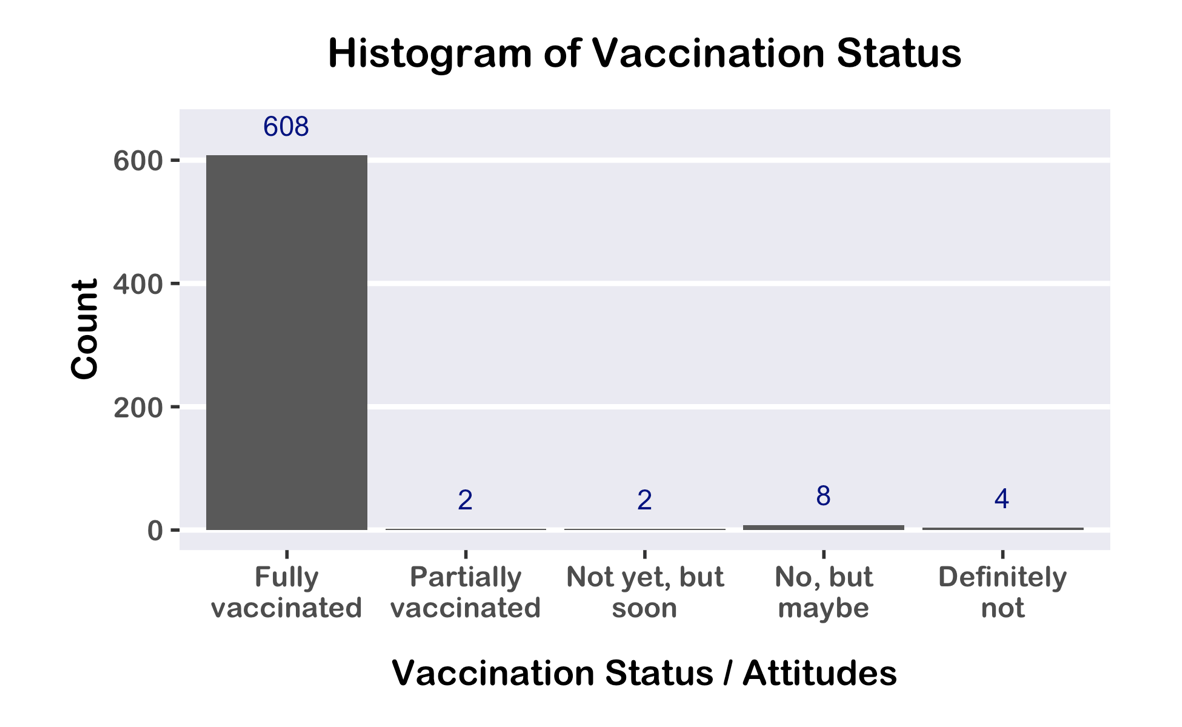

*Figure D.* Histogram of vaccination status / attitudes reported by survey respondents. (Response options are abbreviated for visualization.) The vast majority of survey respondents were fully vaccinated (at least 1 shot of the Johnson & Johnson vaccine or 2 shots of an mRNA vaccine). The survey did not inquire about booster shots. Blue numbers above bars indicate counts.
