# Supplementary material for: Communicating COVID-19 exposure risk with an interactive website counteracts risk misestimation
Source: PLoS One. 2023 Oct 5;18(10):e0290708. doi: 10.1371/journal.pone.0290708 (PMC10553796; doi:10.1371/journal.pone.0290708)
Supplement: S4 Text — (DOCX) [file pone.0290708.s013.docx]

**S4 Text. Variance in Risk Estimation**

In the present study, we used example scenarios (e.g., a coffee shop, a grocery store, or a graduation ceremony) to illustrate different event sizes. We chose to include these scenarios because they help participants visualize the scale of different events, consider risk information in context, and think about exposure risk in everyday life. It is possible that some participants may envision these scenarios in different ways, such as by considering whether an event is indoors or outdoors when assessing risk. This concern disproportionately applies to larger event sizes; for example, a graduation ceremony could take place indoors or outdoors. If these larger event sizes are more ambiguous, leading to greater variation in the interpretations of events, then there should be greater variance in risk estimation for larger event sizes. Contrary to this prediction, we observed that variance in risk estimation was lowest for the largest event size, and approximately the same for the other three event sizes tested in the risk quiz (see figure below). Overall, person-level interpretations may introduce noise, but there does not appear to be any evidence of a systematic bias that can explain the pattern of risk estimation across event sizes.

*
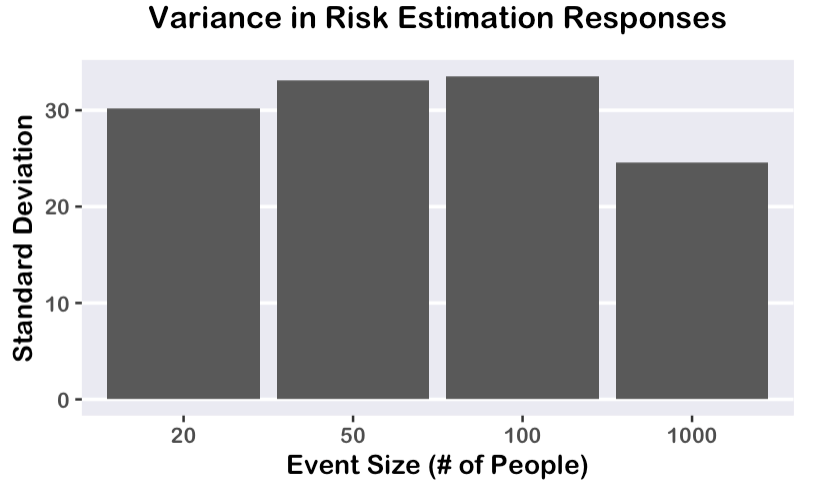
*

**Figure***.* Comparing variance in risk misestimation (standard deviation of risk estimation error) across the four event sizes tested in the risk quiz. We did not observe that larger event sizes were associated with greater variance, countering the concern that larger event scenarios were ambiguous and subject to interpretation.
