# Supplementary material for: Communicating COVID-19 exposure risk with an interactive website counteracts risk misestimation
Source: PLoS One. 2023 Oct 5;18(10):e0290708. doi: 10.1371/journal.pone.0290708 (PMC10553796; doi:10.1371/journal.pone.0290708)
Supplement: S1 File — (PDF) [file pone.0290708.s014.pdf]

License Certificate

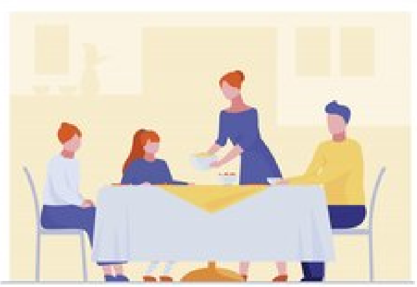

License type:	**Premium license (Unlimited use without attribution) ***
Licensor’s author:	Pch.vector - Freepik.com
Licensee:	appliedbinf
For the item:	Family having dinner
Download date:	2021-07-29
Subscription ID:	2042c520-ef04-492a-8fae-b3b0631d3531 **
Item url:	https://www.freepik.com/free-vector/family-having-dinner_6974884.htm

* as defined in the standard terms and conditions on Freepik.com.

** Agreement valid only upon payment of subscription.

For any queries related to this document or license please contact Freepik Support via **www.freepik.com/profile/support**

Freepik Company, S.L Commercial Registry of Málaga, volume 4994, sheet 217, page number MA-113059, with Tax Number B-93183366 and registered office at 13 Molina Lario Street, 5th floor, 29015 Málaga, Spain.

License Certificate

PREMIUM LICENSE (NO ATTRIBUTION)

If you are a premium user because you have paid for a Subscription the license does not require any attribution

Where you can use Freepik content*:

- Website.
- Software, applications, mobile.
- Printed and digital media (magazines, newspapers, books, cards, labels, CD, DVD, films, television, video, e-mail).
- Advertisement and promotional items.
- Presentation of products and public events.
- Multimedia.
- Decorative (either private or public).

What you CAN DO:

- You have the non-exclusive, non-transferable, non-sublicensable right to use the licensed material an unlimited number of times in any and all media for the commercial or personal purposes listed above.
- You may alter and create derivative works.
- You can use Freepik Content during the rights period, world wide.

What you CANNOT DO:

- Sublicense, sell or rent any contents (or a modified version of them)
- Distribute Freepik Contents unless it has been expressly authorized by Freepik
- Offering Freepik Contents designs (or modified Freepik Contents versions) for download

* The complete content of licenses can be consulted in the Terms of Use, that will prevail over the content of this document.

www.freepik.com/terms_of_use
